# A systematic review on the impact of gestational Lyme disease in humans on the fetus and newborn

**DOI:** 10.1371/journal.pone.0207067

**Published:** 2018-11-12

**Authors:** Lisa A. Waddell, Judy Greig, L. Robbin Lindsay, Alison F. Hinckley, Nicholas H. Ogden

**Affiliations:** 1 National Microbiology Laboratory, Public Health Agency of Canada, Guelph, Ontario, Canada; 2 National Microbiology Laboratory, Public Health Agency of Canada, Winnipeg, Manitoba, Canada; 3 Centers for Disease Control and Prevention, National Center for Emerging and Zoonotic Infectious Diseases, Division of Vector-Borne Diseases, Fort Collins, Colorado, United States of America; 4 National Microbiology Laboratory, Public Health Agency of Canada, Saint-Hyacinthe, Quebec, Canada; University of Toledo College of Medicine and Life Sciences, UNITED STATES

## Abstract

Lyme disease (LD), caused by bacteria of the *Borrelia burgdorferi* sensu lato species complex, is the most common vector-borne disease in North America and Europe. A systematic review (SR) was conducted to summarize the global literature on adverse birth outcomes associated with gestational LD in humans. The SR followed an *a priori* protocol of pretested screening, risk of bias, and data extraction forms. Data were summarized descriptively and random effects meta-analysis (MA) was used where appropriate. The SR identified 45 relevant studies, 29 describing 59 cases reported as gestational LD in the United States, Europe, and Asia (1969–2017). Adverse birth outcomes included spontaneous miscarriage or fetal death (n = 12), newborn death (n = 8), and newborns with an abnormal outcome (e.g. hyperbilirubinemia, respiratory distress and syndactyly) at birth (n = 16). Only one report provided a full case description (clinical manifestations in the mother, negative outcome for the child, and laboratory detection of *B*. *burgdorferi* in the child) that provides some evidence for vertical transmission of *B*. *burgdorferi* that has negative consequences for the fetus. The results of 17 epidemiological studies are included in this SR. Prevalence of adverse birth outcomes in an exposed population (defined by the authors as: gestational LD, history of LD, tick bites or residence in an endemic area) was compared to that in an unexposed population in eight studies and no difference was reported. A meta-analysis of nine studies showed significantly fewer adverse birth outcomes in women reported to have been treated for gestational LD (11%, 95%CI 7–16) compared to those who were not treated during pregnancy (50%, 95%CI 30–70) providing indirect evidence of an association between gestational LD and adverse birth outcomes. Other risk factors investigated; trimester of exposure, length of LD during pregnancy, acute vs. disseminated LD at diagnosis, and symptomatic LD vs. seropositive women with no LD symptoms during pregnancy were not significantly associated with adverse birth outcomes. This SR summarizes evidence from case studies that provide some limited evidence for transplacental transmission of *B*. *burgdorferi*. There was inconsistent evidence for adverse birth outcomes of gestational LD in the epidemiological research, and uncommon adverse outcomes for the fetus may occur as a consequence of gestational LD. The global evidence does not fully characterize the potential impact of gestational LD, and future research that addresses the knowledge gaps may change the findings in this SR. Given the current evidence; prompt diagnosis and treatment of LD during pregnancy is recommended.

## Introduction

Lyme disease (LD), the most common tick-borne disease in North America and Europe is caused by spirochetal bacteria of the *Borrelia burgdorferi* sensu lato species complex (also called *Borreliella*, but referred to herein as *B*. *burgdorferi*) [1]. The most commonly implicated *B*. *burgdorferi* species in human infections include *B*. *burgdorferi* sensu stricto in both North America and Europe and, *B*. *afzelii* and *B*. *garinii* in Europe and Asia [2]. Lyme disease was first recognized in North America in 1975 in the area of Lyme, Connecticut, as a result of an investigation into 51 cases (39 of which were children) that presented with a similar form of arthritis [3]. Early symptoms of infection include a characteristic rash (erythema-migrans, EM), fever, headache, and lethargy. If untreated, the disease may affect the heart, nervous system or manifest as arthritis.

Shortly after its discovery in 1975, the possible effects of gestational LD became an area of research interest given that transplacental infections by other species of spirochetes (e.g. *Treponema pallidum*; relapsing fever *Borrelia* species and *Leptospira interrogans*) are known to occur in several animal species (e.g. dogs, mice, cattle) and in humans [4–8]. The literature on transplacental transmission of *B*. *burgdorferi* in animals is outside the scope of this systematic review (SR). However, some adverse birth outcomes have been recorded for white-footed mice, dogs, cattle, horses, and a coyote. The most common outcomes were reproductive failure (inability to conceive) and fetal loss during pregnancy [9–16]. Animal model experiments identified *B*. *burgdorferi* infection in newborn beagles, indicating that transplacental transmission may occur; however, experiments involving rats, hamsters, and mice have not demonstrated this route of transmission for these species [13,17–19]. Overall, there is some evidence that *B*. *burgdorferi* infection in pregnant animals can result in infection of the newborn, fetal death, and fertility issues [10,12,13].

Given the public health importance of LD and our understanding of other spirochetal diseases, a SR was conducted to identify and summarize the global evidence on “*What is the evidence that gestational Lyme disease in humans causes adverse birth outcomes including congenital abnormalities*?”

## Methods

### Review protocol, team and expertise

This SR was conducted using an *a priori* developed protocol that followed standard SR guidelines [20,21] and the review is reported in accordance with the preferred reporting items for systematic reviews and meta-analysis (PRISMA) statement (S1 Table) [22]. The protocol includes a list of definitions, search algorithms, title/abstract screening form, Risk of Bias tool, and data extraction forms (QA-DE). The protocol (S1 Text), list of relevant included articles (S2 Text) and dataset (S2 Table) are available in the supplementary material.

The review team comprised of individuals with multi-disciplinary expertise in epidemiology, microbiology, entomology, vector-borne diseases, veterinary public health, knowledge synthesis, and information science.

### Search strategy

A pretested search algorithm, found below, was implemented in three bibliographic databases on October 16, 2017: Scopus, PubMed/MEDLINE, and Embase. No limits were placed on the search. The search terms were:

((lyme or borrelia or borreliosis) and (pregnancy or pregnant or maternal or fetus or foetus or newborn or congenital))

The capacity of the electronic search to identify all relevant primary research was verified by hand searching reference lists of three book chapters published between 1995 and 2011 [6,23,24] and three randomly chosen review articles from a list of topic relevant reviews identified during title/abstract screening [25–27]. This process netted 13 citations, conference proceedings, and non-indexed papers that were added to the SR. When omitted citations were no longer being identified the process was stopped. Hand searching of the following websites did not yield additional references:

- Centers for Disease Control and Prevention (CDC) https://www.cdc.gov/
- European Center for Disease Control and Prevention (ECDC) https://ecdc.europa.eu
- Public Health Agency of Canada https://www.canada.ca/en/public-health.html

## Relevance screening and inclusion criteria

References identified by the search were screened for relevance to the review question using a structured and pre-tested form (S1 Text). Those considered relevant to the review question were procured and relevance was confirmed using another pre-tested form implemented prior to proceeding to the risk of bias evaluation. Primary research on humans with gestational LD and any birth outcome (e.g. healthy infants, pregnancy loss, fetal and newborn abnormalities, adverse outcomes or death) were considered relevant to the research question. Chronic Lyme disease was considered to be outside the scope of this review [28]. Global research in any language was included in this SR to minimise language bias. Primary research was defined as original research where authors generated and reported their own data.

### Risk of bias, GRADE and data extraction

Assessment of the risk of bias (RoB) of research relevant to the review question was executed using the whole publication and applying a direct modification of the RoB and Grading of Recommendations Assessment, Development and Evaluation (GRADE) criteria endorsed by the Cochrane collaboration [20,29,30]. The RoB assessment evaluates the internal validity of each study using eight criteria from which the reviewers determine an overall RoB (low, unclear, or high) for each outcome. This informs one of the five GRADE criteria [31]. The data extraction form captures pertinent information and results required for summarization and meta-analysis. Two reviewers (LW and JG) independently assessed the RoB and extracted data on each article.

A GRADE assessment was conducted for each relevant outcome where an outcome is a result from a study; the same outcome may be measured in different studies and as part of the systematic review, outcomes that are alike are grouped together and evaluated as follows. In addition to RoB the other criteria include study design, agreement between studies, precision of results, and evidence of a biological gradient for each outcome. This review included research from any study design; however the less controlled the study, the higher the risk of systematic biases that can result in the study findings deviating from the truth. The GRADE framework prescribes a gradually lower GRADE as the risk of bias increases [32]. This means that randomized controlled trials and well-designed cohort studies could be graded moderate/high (*** or ****), whereas case control studies and cross sectional studies are likely to be graded as low (**) and case reports and expert opinion receive a very low grade (*). Across each outcome/study design pair, groups of similar studies would be evaluated for overall RoB, agreement, precision and evidence of a biological gradient, which could result in up-grading (or down-grading) the level of confidence in the evidence for that outcome [32,33].

The final GRADE is assessed considering all five GRADE criteria for each unique outcome to indicate the level of confidence in the evidence [30]. The one to four star grading system indicates: **** high confidence that the effect estimate is close to the true effect; *** moderate confidence in the effect estimate, but future studies may be substantially different; ** limited confidence in the effect estimate, the true effect may be substantially different; * very little confidence in the effect estimate, the true effect is likely to be substantially different [32–34].

### Systematic review management and analysis

Search results were imported into reference management software (Endnote X7, Thomson Reuters, USA), duplicates were removed and the list of unique citations was imported into a web-based electronic SR management platform (DistillerSR, Evidence Partners, Ottawa, Canada). All stages of the SR were conducted within this software and collected data were exported into Excel spreadsheets (Microsoft Corporation, Redmond, WA), organised (sub grouped by common exposure and outcome) and summarized (frequencies and percentages).

Post-hoc calculations such as computing unadjusted odds ratios (OR) from contingency table data were done, where necessary, to ensure comparability of the data. Whenever studies reported both unadjusted and adjusted OR measures, the adjusted measure was selected for inclusion in the tables and/or meta-analysis model [20].

Random-effects meta-analysis using the Der Simonian and Laird method was conducted for each unique group of studies if sufficient data were available (ie: if there were ≥2 studies, and the studies were comparable) [35]. For meta-analysis of proportions, the Freeman-Tukey double arcsine transformation was used to stabilize the variances [36]. Heterogeneity was measured using I^2^, which indicates the proportion of variation in the measures of association across studies due to heterogeneity rather than sampling error [37]. It was not possible to test for publication bias due to a limited number of studies (<10) in any subgroup [38].

## Results

**Fig 1:**
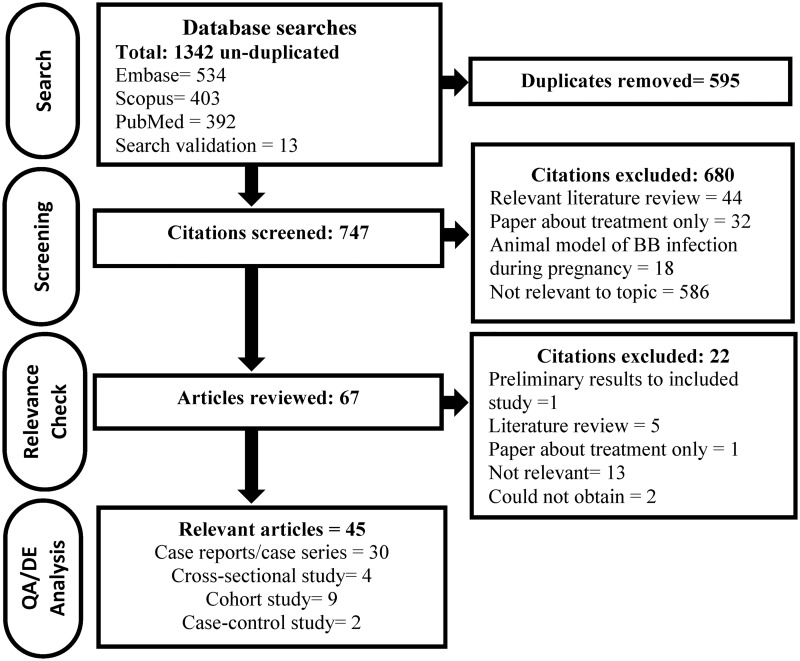
The flow of citations and research papers through the systematic review process.

Forty-five relevant primary research studies were identified after screening 746 unique citations and 67 full papers (Fig 1). Thirty-five of these papers were published in English, two in Czech, two in Serbian and one each in French, Italian, Dutch, German, Russian, and Polish. Despite efforts to minimise bias by including all available research, we were unable to procure two potentially relevant articles [39,40].

**Fig 2:**
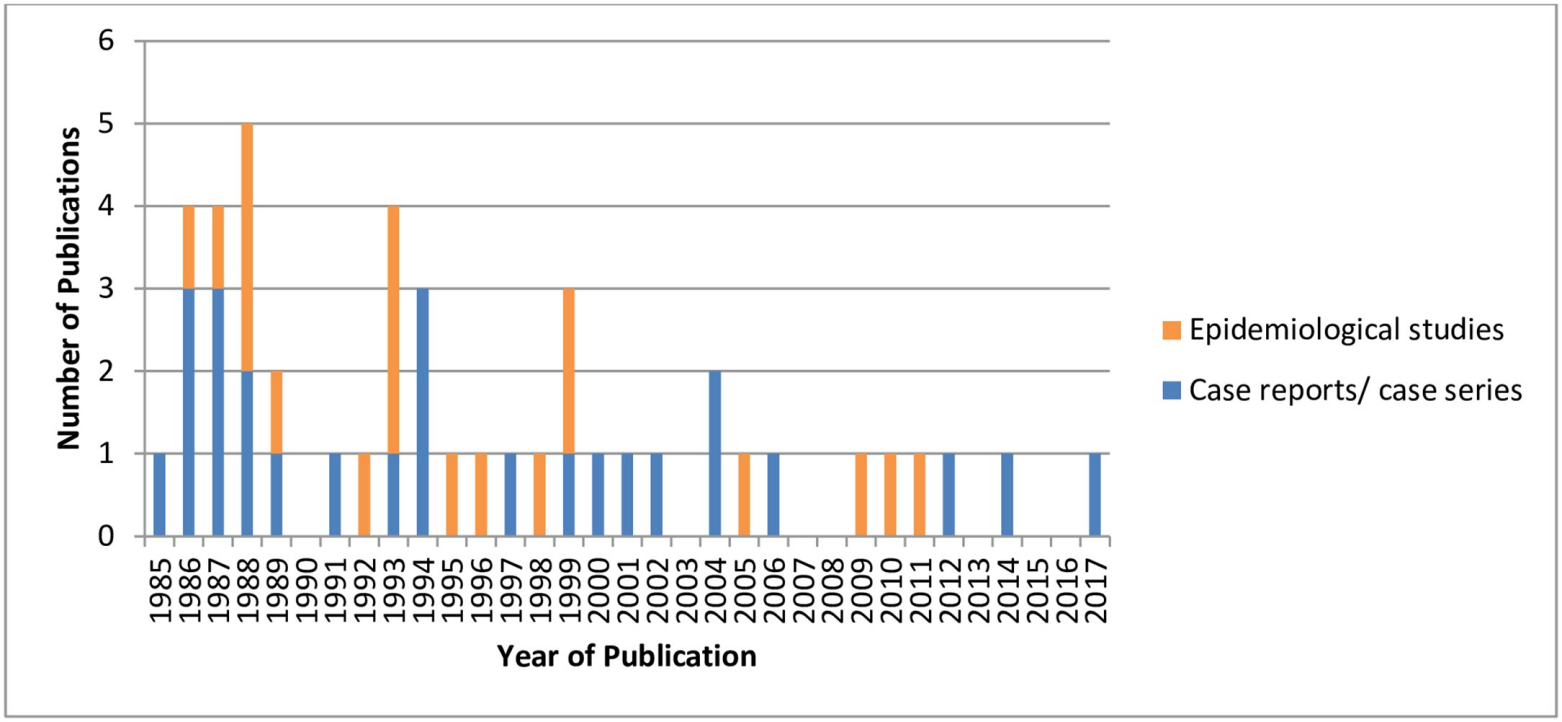
The distribution of publication dates of 45 primary research publications relevant to the impact of gestational Lyme disease included in this systematic review grouped by studies that had epidemiological data or case report data.

The literature on adverse birth outcomes associated with gestational LD is based on case reports and case series (n = 26), case series with epidemiological data (n = 4), and epidemiological studies (n = 15) (Fig 1). The case report and case series studies reported detailed information on one or more cases of gestational LD and the pregnancy and/or birth outcomes of the case. The case series with epidemiological data provided information on a group of gestational LD cases and associations between possible risk factors (e.g. untreated vs. treated LD) and the risk of an adverse birth outcome. The cohort, cross-sectional, and case control studies investigated possible differences in the frequency of adverse outcomes between an exposed and unexposed control group. Case studies captured in this review were published between 1985 and 2017, whereas the epidemiological studies were published between 1986 and 2011 (Fig 2). Across studies, exposure was defined as: evidence of clinical manifestations of LD during pregnancy, serological evidence of LD during pregnancy or surrogate measures of exposure to LD (e.g. history of tick bites or living in a geographic area considered endemic for LD).

Each study was evaluated for its RoB, which evaluates how well the study was conducted. Many criteria for RoB assessment do not apply to case reports, thus case report assessment focused on complete reporting. There were 26 case reports or case series included in this SR and three had an “unclear” RoB designation because the diagnostic tests performed or outcomes were not reported in sufficient detail (Table 1). The RoB for 19 epidemiological studies was 42% low, 42% unclear and 16% high (Table 1). Studies with an “unclear” RoB had one or more criteria that could not be assessed because the required information was not reported including: i) missing information on the blinding of patients and/or outcome assessors, or ii) unexplained loss to follow-up or loss of observations. “High” RoB studies had several flaws in the research process that may bias the results including failure to account for, or examine, important confounders or other biases. Insufficient information to assess RoB criteria is likely a reporting issue in many papers but results in an “unclear” or “high” RoB classification depending on the cumulative deficiencies of the study. The RoB evaluation for each study is available in S2 Table.

**Table 1:** General characteristics of 45 included primary research publications.

Category		Count
**Continent**^1^		
**North America**		19
**Europe**		24
**Asia**		3
**Outcomes Reported**^1^		
**Maternal outcome**		37
**Miscarriage/ pregnancy loss**		13
**Fetal outcome**		8
**Newborn outcome**		35
**Infant/child outcomes**		5
**Study design**	**Risk of Bias (RoB) Assessment**	
**Case study/ case series**		**26**
	Low RoB	23
	Unclear RoB	3
**Case series** ^2^		**4**
	Low RoB	1
	Unclear RoB	2
	High RoB	1
**Case control**		**2**
	Low RoB	1
	Unclear RoB	1
**Cross sectional**		**4**
	Low RoB	2
	Unclear RoB	2
**Cohort**		**9**
	Low RoB	4
	Unclear RoB	3
	High RoB	2

^1^ Total number sums to >45 as studies can fall into more than one category.

^2^ Case series with epidemiological data.

### Case reports of gestational Lyme disease

Details of 59 cases were summarized in 29 publications from the USA, Europe, and Asia describing gestational LD and pregnancy outcomes between 1969 and 2017, Table 2. These case reports and case series received a GRADE of *, indicating that future evidence may be inconsistent with the conclusions of these studies. Across 59 cases, negative outcomes for the fetus or newborn occurred in 36 (61%) pregnancies. Negative outcomes ranged from spontaneous miscarriage (termination of pregnancy prior to when the fetus is considered viable, approximately 28weeks) (n = 10), fetal death and stillbirth after 28 weeks (n = 2) and death shortly following birth (n = 8, four were premature, born before 36 weeks gestation), to a range of congenital abnormalities and health issues (n = 16) including hyperbilirubinemia, respiratory distress, syndactyly, and ureter and heart abnormalities, Table 2. For six infants, a wide range of long term conditions were reported [41–45], Table 2. Healthy children (including one set of twins) were born in 23 pregnancies from mothers who had clinical manifestations (n = 10), serological evidence (n = 3) or both (n = 10) consistent with LD.

**Table 2:** Summary of pregnancy outcomes from 59 case reports diagnosed with gestational Lyme disease and test results for direct detection of *B*. *burgdorferi* or spirochetes or antibodies against the agent of Lyme disease.

Case Data							Test results by sample taken, test results are noted by +/- and positive results are shaded									Detailed Findings
							Mother Clinical and Test Results			Cord Blood	Placenta	Fetus/ Infant Autopsy	Newborn Samples		Child Samples	
Ref	Country/ State	Year	Age	LD Trimester^1^	LD Treated?	Week Pregnancy Ended	Clinical	Serology	Sample Culture	Serology	Tissue	Tissue	Serology	Tissue	serology	
**North America -USA only**																
[46]	Arkansas (treated in Germany)	1997	34	2^nd^ ^5^	Yes	term	FP	2-tier IgM+ ^6^		S IgG-, IgM-	PCR -					Healthy twins. 2-tier IgM only: IFA+, IB + Mother tested at approx. 31 weeks pregnant using European protocol.
[47]	California	1987	NR	NR	No	term	AR	ELISA-				WS+, culture+				Newborn died at 8 days with Peripheral cyanosis, systemic hypertension, metabolic acidosis, myocardial dysfunction and abdominal aortic thrombosis. Spirochete appeared similar to the original Long Island tick isolate (*B*. *burgdorferi* questionable), cultured from brain; brain and heart positive by staining. Timing of mother’s test is unknown.
[48]	New Jersey	1991^2^	NR	2^nd^	Yes	term	EM	IB IgM+, IgG+								Healthy newborn Mother tested at 27 weeks pregnant.
[49]	New York	1978	25	unknown	No	39	None					IHC+				Newborn died at 4 hours. Multiple anomalies: large ventricular septal defect, hydrocephalus, omphalocele, clubfoot, spina bifida, and meningomye locele. Spirochetes in fetal tissue.
[49]	New York	1979	33	unknown	No	40	None					IF+				Newborn died at 30 minutes. Spirochetal fragments in fetal tissue.
[49–51]	New York	1985	24	1^st^ ^5^	No	term	NR	IFA+, ELISA+^3^			DF+, MA+	DF+, IF+, K+, MA+				Stillborn. Dark field microscopy showed *B*. *burgdorferi* in the liver, adrenal, brain, heart, and placenta. Cultured spirochetes from liver. Lack of tissue inflammation noted. Mother tested postpartum.
[49]	New York	1985	26	unknown	No	37	NR				WS+					Newborn developed respiratory distress, treated with antibiotics and recovered.
[49]	New York	1986	19	unknown	No	term	NR				WS+					Newborn developed respiratory distress, treated with antibiotics and recovered.
[49]	New York	1986	28	2^nd^	Yes	term	EM	IFA-, ELISA-		IFA-, ELISA-	BSK-H+ WS+					Healthy newborn Mother appears to have acquired LD 2x during pregnancy and treated 2x. Mother tested at delivery.
[49]	New York	1986	23	2^nd^	Yes	term	EM	S-			WS-					Healthy newborn Mother tested in 2^nd^ and 3^rd^ trimester.
[49]	New York	1986	34	unknown	No	17	None	S-				IF+				Fetal death. Spirochetes found in brain. Mother tested postpartum.
[49]	New York	1986	25	unknown	No	12	NR					BSK-H+, IHC-				Fetal death. *B*. *burgdorferi* isolated from fetal kidney
[49]	New York	1988	21	unknown	No	16	None	S-				IHC+, MA+				Fetal death. Spirochetes isolated in brain. Mother tested postpartum.
[49,51]	New York	1989^2^	22	unknown	No	19	None	S-				IF+				Fetal death. Coarctation of the aorta. No inflammation in spirochete positive tissues (not specified) was noted. Mother tested postpartum.
[49,51]	New York	1989^2^	37	unknown	No	23	None	S-				IF+				Fetal death. Spirochetes found in the kidney. No inflammation in spirochete positive tissues. Mother tested postpartum.
[49,51]	New York	1989^2^	32	unknown	No	15	None	S-			IF+	IF+				Fetal death. Spirochetes found in the liver and placenta. Mother tested postpartum.
[49]	New York	1989^2^	27	unknown	No	25	AR					IF+				Fetal death, large intraventricular septal defect detected. Positive tissue not described.
[27]	New York	2007^2^	42	3^rd^	Yes	41	AR	2-tier ^6^ IgM+, IgG+	PCR+	S IgG+, IgM-	WS-, GM-					Healthy newborn Mother’s synovial fluid PCR+ post-partum (after 1^st^ round of treatment). 2-tier: ELISA+, WB IgM+/IgG+ Mother tested at 34 weeks pregnant.
[52]	Wisconsin	1984	28	1^st^	No	35	EM, AR	IFA IgG+				D+				Newborn died at 39 hours. Congenital hypoplastic left heart complex malformation. Spirochetes, morphologically compatible with *B*. *burgdorferi*, found in spleen, renal tubes and bone marrow. No inflammation in spirochete positive tissues. Mother tested postpartum.
[44]	USA- not specified	1986^2^	34	1^st^	Yes	20	EM, AR	S+			IF-, culture-	IF-, culture -				Fetal death. Mother tested at 8 and 16 weeks pregnant
[44]	USA- not specified	1986^2^	32	1^st^	No	36	FP, AR									Newborn with hyperbilirubinemia, recovered.
[44]	USA- not specified	1986^2^	30	2^nd^	Yes	term	EM, AR									Healthy newborn except for syndactyly
[44]	USA- not specified	1986^2^	31	2^nd^	Yes	term	EM						S-			Healthy newborn. At 8 months child was diagnosed with cortical blindness and developmental delay. Infant tested at 1 year.
[44]	USA- not specified	1986^2^	31	3^rd^	No	term	EM, MG									Healthy newborn except for a generalized, petechial, vesicular rash and hyperbilirubinemia
[53]	USA- not specified	2017^2^	31	2^nd^	Yes	41	EM	ELISA+								Healthy newborn Mother tested at 16 weeks pregnant.
**Europe**																
[42]	Austria	1969	NR	Pre ^5^	No	NR	EM								IB+	New born had several minor abnormalities (huge sacral hemangioma, gluteal atrophy). Samples taken 20 years later for serological testing, whole family was positive.
[54]	Czech Republic	1986	26	2^nd^	Yes	32	EM				NR-		S-			Premature newborn with respiratory distress syndrome and anemia at birth. Time of newborn test is not reported.
[54]	Czech Republic	1986	24	3^rd^ ^5^	NR	41	EM						S-			Healthy newborn Time of newborn test is not reported.
[55]	Denmark	1987^2^	29	3^rd^ ^5^	Yes	39	EM	S+		S-						Healthy newborn Mother tested at 36 weeks pregnant.
[56]	France	1994^2^	27	3^rd^ ^5^	Yes	term	EM	ELISA IgG+, IgM+		ELISA IgG-, IgM-			ELISA IgG-, IgM-			Healthy newborn Mother tested at delivery. Newborn tested at 6 and 9 months.
[45]	Germany	1981	NR	unknown	No	37	none	ELISA IgG+ ^4^							ELISA IgG+ ^4^	Child had many conditions: intellectually retarded, deaf, enlarged head, fontanelle 4x3 at 4 years, chronic meningitis, protruding eyes, blepharitis, conjunctivitis, strabismus, maculopaplar rash, pruritus, recurrent arthritis of the knees, adenomegaly, hepatosplenomegaly. Serology done retrospectively when child was 4 years old. There is no clinical history of LD before or during pregnancy in the mother. Time of mother’s test is not reported.
[57,58]	Germany	1984	37	1^st^ ^5^	Yes	term	EM	ELISA IgM+ IgG-, IHA+, IFA-				WS+, MA+				Newborn died at 23 hours due to prenatal brain damage. Spirochetes identified in the brain and liver. Mother’s 1984 serology samples were negative with 1984 tests and positive with 1986 tests.
[58]	Germany	1986^2^	NR	3^rd^	NR	term	EM						S-			Healthy newborn Newborn tested more than once between birth and 9 months.
[59]	Germany	1994^2^	36	3^rd^	Yes- post	term	AR	S IgG-, IgM+					S IgG-, IgM-			Healthy newborn. Mother had joint pain, IgM+ at day 28 of symptoms, at day 42 IgM- and IgG-. Newborn tested at birth.
[60]	Italy	1993	32	unknown	No	39	None	IFA IgG+			WS-			WS+, PCR+	**9 m**: ELISA, IFA, WB IgG- & IgM-**13m**: ELISA-, IFA-WB IgG+, IgM -	Healthy newborn. At 3 weeks: multiple annular erythemas and fever, relapsed at 9, 12, 24 and 36 months. Each episode between 9 and 36 months treated with antibiotics. 13 month IgG western blot was positive for *B*. *garinii* 41 kD, 30 kD and 61 kD. 36 months a borrelia-like organism failed to culture on BSK-H. Mother was tested postpartum.
[61]	Poland	2001	25	3^rd^	Yes	term	EM	ELISA IgM+, IgG+								Newborn had hyperbilirubinemia and recovered Mother tested approx. 25 weeks pregnant.
[62]	Poland	2012^2^	30	3^rd^	Yes	term	EM	2 -tier IgM+, IgG-					ELISA-			Healthy newborn 2-tier: ELISA IgM+ & WB IgM+ Mother tested postpartum & post treatment. Newborn tested after birth.
[63]	The Netherlands	2000^2^	33	Pre	Yes	38	FP	S IgM+/IgG+								Healthy newborn. Mother tested at 16, 17 (IgM+ only) and 19 (IgM+ and IgG+) weeks pregnant.
[64]	The Netherlands	2002^2^	37	3^rd^ ^5^	Yes	term	EM, FP	2-tier ^6^ IgM+, IgG+					ELISA IgM-			Healthy newborn 2-tier: ELISA IgM+, IgG + & WB IgM+, IgG + Mother tested at 37 weeks pregnant. Newborn tested at birth.
[65]	Serbia	1991	25	1^st^ ^5^	Yes	term	EM	S IgG-, IgM-								Healthy newborn Mother tested at 10 weeks pregnant.
[66]	Serbia	1992	29	2^nd^ ^5^	NR	term	NR	IFA IgM+, IgG+								Healthy newborn Mother tested at 25 weeks pregnant.
[66]	Serbia	1992	25	1^st^ ^5^	NR	term	NS	IFA IgM+, IgG+								Healthy newborn Mother tested at 18 weeks pregnant.
[43]	Slovenia	1986	33	NR	Yes—post	34	None	IFA IgG+				DF+				Fetal death. Spirochetes seen in lung, liver and brain tissue. Mother tested postpartum: IFA IgG+, 6 months after treatment IgG-
[43]	Slovenia	1999^2^	26	1^st^ ^5^	Yes	32	EM	S+				DF+				Died within hours of birth. Hydrocephalus, a fluidothorax, ascites, no malformations. Spirochetes in lung and liver tissue. Mother tested at the end of the second trimester.
[43]	Slovenia	1999^2^	26	1^st^ ^5^	Yes	25	EM	IFA-				WS-				Premature newborn died within minutes of birth. Autopsy: chorioamnionitis and vasculitis of umbilical vessels Mother tested at 6 weeks pregnant.
[43]	Slovenia	1999^2^	25	1^st^ ^5^	Yes	25	EM	IFA-				WS-				Premature newborn died immediately, no relevant findings on autopsy. Mother tested at 11 weeks pregnant.
[43]	Slovenia	1999^2^	26	2^nd^ ^5^	Yes	33	EM	IFA-								Premature newborn had severe hyperbilirubinemia, staphylococcal infection and apnoea. Mother tested at 7 weeks pregnant.
[43]	Slovenia	1999^2^	23	2^nd^ ^5^	Yes	26	EM	IFA-								Premature newborn with respiratory distress syndrome, bilateral ventricular and periventricular bleeding, intraverebral parieto occipital bleeding. Child recovered. Mother tested at 21 weeks pregnant.
[43]	Slovenia	1999^2^	31	2^nd^ ^5^	Yes	36	EM	IFA-								Healthy newborn Mother tested at 26 weeks pregnant.
[43]	Slovenia	1999^2^	27	1^st^ ^5^	Yes	36	EM	IFA-								Newborn had respiratory distress, a wet lung and later pneumothorax and atelectasis. Mother tested at 16 weeks pregnant.
[43]	Slovenia	1999^2^	28	3^rd^	Yes	40	EM	IFA-								Healthy newborn. 7 months old bilateral ureteral stenosis with hydronephrosis identified. Mother tested at 29 weeks pregnant
[43]	Slovenia	1999^2^	23	3^rd^ ^5^	Yes	term	EM	IFA-								Healthy newborn, 5 months old vesicoureteral reflux was diagnosed. Mother tested at 33 weeks pregnant.
[43]	Slovenia	1999^2^	29	1^st^	Yes	term	EM	IFA-								Healthy newborn, 10 months old unilateral ureteral stenosis and hydroureter. Mother tested at 16 weeks pregnant.
[43]	Slovenia	1999^2^	37	1^st^ ^5^	Yes	term	EM	IFA-								Healthy newborn with syndactyly Mother tested at 6 weeks pregnant.
[43]	Slovenia	1999^2^	28	1^st^	Yes	9	EM	IFA-								Spontaneous abortion 9 weeks. Mother tested at 7 weeks pregnant.
[43]	Slovenia	1999^2^	23	1^st^	Yes	10	EM	IFA-								Spontaneous abortion 10 weeks. Mother tested at 5 weeks pregnant.
[41]	Spain	2014^2^	31	2^nd^	Yes	36	NS		BSK-H+, PCR+ ^6^, RLBH+							Child developed cholelithiasis in utero (diagnosed at 26 weeks), at 2.5 years still suffered from the condition. Positive cell culture for *Borrelia* ssp. from unknown sample, confirmed LD by PCR and RLBH. Mother tested at 20 weeks pregnant.
**Asia**																
[67]	Turkey	2005^2^	18	unknown	No	36	None	S IgM+, WB IgG+					WB IgM equivocal			Newborn had congenital triventricular hydrocephalus and aquaductus cerebri stenosis, transepandymal cerebrospinal-fluid leakage. Mother WB IgG: 31 kDa & Newborn WB IgM: 41 kDa and 75 kDa Mother tested at 34 weeks pregnant. Newborn tested at birth.
[68]	Russia	1999	21	2^nd^	Yes	term	NS	IFA IgG+								Healthy newborn Mother tested at 20 weeks pregnant.

^1^ LD trimester = the trimester that LD was acquired by the case, options "pre" before pregnancy, 1st trimester is pregnancy weeks 1–12, 2nd trimester is pregnancy weeks 13–27 and 3rd trimester is pregnancy weeks 27 to 40+ weeks, "post" after pregnancy.

^2^ Case date not provided, publication date used.

^3^ Conflicting postpartum serological results: CDC and New York State Department of Health found strongly reactive results by IFA and ELISA, however Dr. Allen Steere did not detect specific antibodies for *B*. *burgdorferi*.

^4^ ELISA test conducted on mother’s serum over 4 years after the birth of her child. Child’s serum was tested (unknown test) at unreported times between birth and 4 years old.

^5^ Tick bite reported by mother.

^6^ Indicates cases in which *B*. *burgdorferi* infection in the mother was detected using laboratory methods recommended by current guidelines.

NR = not reported, NS = non-specific,

EM = erythema migrans rash associated with Lyme disease. FP = facial palsy, AR = arthritis, MG = meningitis.

S = serological test not described. ELISA = enzyme-linked immunosorbent assay. IHA = indirect hemagglutination. IFA = indirect immunofluorescence assay. IB = immunoblot, 2-tier = 2-tier testing indicates a positive or equivocal ELISA or IFA followed by a confirmatory western blot. IgG/ IgM = Immunoglobulin G and M are indicated for serology where described.

BSK-H = culture Barbour Stoenner Kelly II medium. K = Kelly's medium. PCR = polymerase chain reaction. RLBH = reverse line blot hybridization assay. WS = Warthin starry silver stain, GM = Gomori methenamine stain. D = Dieterle staining method. IF = indirect immunofluorescence DF = Dark field microscopy, not further specified. MA = specific monoclonal antibody H5332. IHC = immunohistochemistry.

Laboratory testing of the newborn or fetus was not reported in 28 cases, Table 3, and for these the possible role of LD was determined by evidence in the mother (clinical manifestations [n = 13], diagnostic test results [n = 3], or both [n = 10]), while for 2 cases the only evidence of infection was identification of spirochetes in the placenta reported to be *B*. *burgdorferi*, Table 2. The newborn and fetus cases with laboratory evidence of infection (n = 31), included serological test results (n = 13) of which only two were considered positive and one was borderline, Table 3. Fetal or newborn tissue samples (n = 19) were examined and *B*. *burgdorferi* was identified in 17 cases using staining, indirect immunofluorescence (IF), or PCR to confirm the presence of *B*. *burgdorferi*. Not all tests used in the studies are considered reliable; those considered reliable include PCR with specific primers, IF using specific antibodies, and culture when it is confirmed by IF or PCR (n = 12) [69–71]. Direct microscopic detection (n = 7) of *B*. *burgdorferi* (using bacteria staining and dark field microscopy) is generally considered of limited value in diagnosis due to both the small number of spirochetes seen and the risk of false positive results for a range of reasons reviewed elsewhere [72]. Across the case reports the specificity of the primers used for PCR and methods of culture confirmation were often not mentioned, but we included these reports nevertheless. Evidence of infection in the placenta (presence of spirochetes) and/or cord blood (presence of antibody) was sought in 12/59 cases, Table 3. Of these 5/11 placentas and 1/5 cord blood samples were positive (for IgG antibodies only), two of the placenta positive results were based on the results of indirect immunofluorescence. In the positive cord blood case and one placenta-positive case the child was healthy at birth.

**Table 3:** An overview of the features of 59 case reports diagnosed with gestational Lyme disease.

Case characteristic	Positive / total cases	Not reported/ not done
**Pregnant Women**		
**Pregnant women with clinical manifestations of LD**	41/54	5
**Pregnant women with laboratory test results**	23/33	26
**Pregnant women with test results from currently recommended laboratory tests**^1^	4/4	N/A
**Pregnant women where clinical symptoms were not reported (n = 2) or not specific (n = 8), but laboratory test results were reported**^2^	7/10	N/A
**Other samples tested**		
**Spirochetes detected in placenta**	5/11	N/A
**Cord blood serology**	1/5	N/A
**Fetus, Newborn or Child**		
**Any test result for a fetus, newborn or child**	18/31	28
** Spirochete identified in tissue collected at autopsy**	15/18	2
** Spirochetes identified following autopsy conducted on fetus from pregnant women not diagnosed with gestational LD**.	5/5	N/A
** Spirochete identified in tissue sample from a live child**	1/1	N/A
**Serology results in the newborn or child**	2/13	34
**Frequency of Negative Birth Outcomes**		
**1^st^ trimester miscarriage**	3/59	N/A
**2^nd^ trimester miscarriage**	7/59	N/A
**3^rd^ trimester fetal death/ stillbirth**	2/59	N/A
**Death shortly after birth**	8/59	N/A
**Abnormalities/ health issues**^3^	16/59	N/A
** Long term conditions**	6/16	N/A
**Healthy Infants**	23 (1 set of twins)/59	N/A

^1^ A subset of total laboratory tests, evaluated based on laboratory methods recommended by current guidelines [70,71]

^2^ Spirochetes identified in placenta (n = 3) &/or fetal tissue (n = 3)

^3^ Examples of adverse outcomes: hyperbilirubinemia, respiratory distress, syndactyly, and ureter and heart abnormalities

Amongst the 59 pregnancies identified in the case studies in this SR, 33 (56%) pregnant women were tested for LD; but diagnostic methods currently considered reliable (direct detection methods as described above or the two-tier EIA followed by the Western Blot) were used in only four cases, Table 3 [27,41,46,64,69,72,73]. In five other cases, the mother was not diagnosed with LD by clinical symptoms or a diagnostic test, but instead was considered retrospectively to have suffered from LD when the cause of fetal or newborn demise was investigated and possible *B*. *burgdorferi* spirochetes were identified in the placenta (n = 3) and/or fetal tissue (n = 3) [49,51].

There was treatment information for 19/23 pregnancies that resulted in healthy newborns; 18/19 (95%) were treated for LD during pregnancy and subsequently had no adverse birth outcomes. Healthy newborns were born to mothers that had a wide range of gestational LD symptoms from EM to neuroborreliosis and LD was acquired in all trimesters (Table 2). In contrast, 34/36 pregnancies have treatment information and a negative birth outcome, of these only 14/34 (41%) were treated during pregnancy. Among the 20 cases that were not treated and had negative birth outcomes, 10 occurred in mothers with no clinical history of symptoms consistent with LD according to current guidelines [70,71]. The other 10 untreated cases were mainly diagnosed retrospectively or after parturition, so there was no opportunity for treatment during pregnancy.

Across cases, evidence that transplacental transmission of *B*. *burgdorferi* can occur was shown by testing the placenta (n = 11) and deceased fetal/newborn tissue (n = 18), Table 3. Adverse birth outcomes occurred in 4/5 placenta positive cases (2 stillbirths and 2 cases of respiratory distress that recovered), in 2/6 placenta-negative cases (one premature birth and one case reported as relapsing LD beginning at 3 months of age, and spirochetes were identified in one or more fetal tissues in 15/18 autopsies (Table 2). Only one case (in Germany) described the full range of expected observations (clinical manifestations in the mother, negative outcome for the child, and laboratory detection of *B*. *burgdorferi* in the child) that would give confidence that vertical transmission of *B*. *burgdorferi*, with negative consequences for the fetus, occurs [57,58]. The reports from the autopsies (n = 18) did not provide an explanation for how the presence of *B*. *burgdorferi* was associated with the pathology seen in the fetus [49,51,52,57,58]. A common autopsy observation was the lack of inflammation or immune response against *B*. *burgdorferi* infection in the fetus. Across all cases there were no consistent clinical outcomes resulting from gestational LD, and a linkage between fetal loss and gestational LD remains unclear from the case reports.

### Epidemiological studies

There are 19 epidemiological studies identified in this review, which include nine cohort studies, four cross sectional studies, two case control studies, and four case series. The studies were conducted in the USA (n = 10) and Europe (n = 9) and published between 1986 and 2011. One cohort did not have extractable epidemiological outcomes, but the data from the pregnant LD cases are included in the previous section [66]. A second study is not included in this review because there were no extractable outcomes [74]. This study also received a very high RoB evaluation due to incomplete reporting of methods and outcomes, lack of blinding, failure to account for or examine important confounders, and other biases including the potential of funding bias [74].

#### Adverse outcomes in LD exposed vs unexposed populations

Eight studies reported differences in prevalence of one or more types of adverse birth outcomes in exposed compared to unexposed populations, Table 4. The definition of ‘exposed’ in these studies included women diagnosed with gestational LD during the study, a history of LD (based on clinical chart review), positive LD serology during pregnancy, or those considered to be at higher risk of LD (measured indirectly by having a history of tick bites or living in a known endemic area for LD risk). Adverse birth outcomes amongst and within the epidemiological studies varied widely and included very common outcomes such as preterm birth and hyperbilirubinemia [75,76], as well as less frequent and more serious major congenital malformations. Some studies reported all adverse outcomes together without additional details by type of outcome (Table 4). Meta-analysis was considered inappropriate for all outcomes due to the variability in study design, definition of LD, and range of adverse outcomes.

**Table 4:** Measures of association extracted from eight studies on adverse birth outcomes and LD during or before pregnancy, positive LD serology during pregnancy, and surrogate measures of possible exposure to LD (e.g. tick bites or living in an endemic area). (Significant odds ratios are bolded in the results.).

Ref	Study	Adverse outcome definition	Diagnosis of LD or surrogate measure of exposure in mothers	OR*	95% Conf. Interval		N
Association with adverse birth outcomes due to having a positive LD serological test during pregnancy or history of gestational LD (GRADE ***)							
[79]	Carlomagno (1988)	spontaneous miscarriage	Serological screening only (IgG) ^2^^,^ ^3^	2.14	0.50	9.09	98
[77]	Strobino (1993)	spontaneous miscarriage	Serological screening (IgG or IgM) ^2^^,^ ^4^^,^ ^9^ and clinical history of LD	0.49	0.03	8.41	1521
[77]	Strobino (1993)	spontaneous miscarriage	Clinical gestational LD ^10^	0.39	0.03	6.01	1746
[77]	Strobino (1993)	spontaneous miscarriage	LD <1 year before conception	1.73	0.69	4.36	1760
[77]	Strobino (1993)	spontaneous miscarriage	LD >1 year before conception	1.20	0.32	4.51	1752
[78]	Dlesk (1989)	spontaneous miscarriage	Serological screening only (IgG or IgM)^2^^,^ ^3^^,^ ^9^	0.71	0.08	5.93	126
[77]	Strobino (1993)	history of miscarriage	Serological screening (IgG or IgM) ^2^^,^ ^4^^,^ ^9^ and clinical history	0.86	0.19	4.00	1521
[80]	Bracero (1992)	premature rupture of membranes	Serological screening only (IgG or IgM)^2^^,^^3^^,^ ^9^	1.01	0.12	8.88	134
[80]	Bracero (1992)	premature labour	Serological screening only (IgG or IgM) ^2^^,^^3^^,^ ^9^	1.46	0.16	13.10	134
[80]	Bracero (1992)	low birth weight	Serological screening only (IgG or IgM) ^2^^,^^3^^,^ ^9^	2.27	0.41	12.58	134
[80]	Bracero (1992)	apgar <7	Serological screening only (IgG or IgM) ^2^^,^^3^^,^ ^9^	3.36	0.35	32.54	134
[80]	Bracero (1992)	small for gestational age	Serological screening only (IgG or IgM) ^2^^,^^3^^,^ ^9^	6.89	0.62	76.46	134
[80]	Bracero (1992)	congenital abnormality, all^1^	Serological screening only (IgG or IgM) ^2^^,^^3^^,^ ^9^	5.62	0.21	150.06	134
[81]	Strobino (1999)	congenital cardiac abnormality	History of LD ^10^	0.85^+^^ǂ^	0.39	1.89	1500
[77]	Strobino (1993)	congenital abnormality, all^1^	Clinical gestational LD^10^	0.53^ǂ^	0.07	4.16	1521
[77]	Strobino (1993)	congenital abnormality, all^1^	LD <1 year before conception	1.65^ǂ^	0.60	4.57	1760
[77]	Strobino (1993)	congenital abnormality, all^1^	LD > 1 year before conception	2.94^ǂ^	0.98	8.86	1752
[77]	Strobino (1993)	congenital abnormality, minor^1^	Clinical gestational LD^10^	0.80^ǂ^	0.10	6.28	1521
[82]	Williams (1995)	congenital abnormality, major ^1^	LD before pregnancy	3.26	0.75	14.20	2386
[82]	Williams (1995)	congenital abnormality, all^1^	LD before pregnancy	1.13	0.26	4.85	2386
[82]	Williams (1995)	congenital abnormality, major ^1^	Clinical gestational LD^10^	6.80	0.78	59.00	2386
[82]	Williams (1995)	congenital abnormality, all^1^	Clinical gestational LD^10^	2.37	0.28	20.42	2386
Association with adverse birth outcomes and an IgG or IgM positive cord blood serological test (GRADE **)							
[83]	Williams (1988)	adverse birth outcomes	Cord blood serology (IgG) ^5^	0.40	0.05	3.07	255
[82]	Williams (1995)	congenital abnormality, minor^1^	Cord blood serology (IgG) ^5^	0.63	0.08	4.70	2386
[84]	Lakos (2010)	adverse birth outcomes	Cord blood serology (IgG)^6^	No est.			74
Association with congenital abnormalities and tick bites during pregnancy (GRADE **)							
[82]	Williams (1995)	congenital abnormality, all^1^	Tick bite during pregnancy	1.63	0.77	3.47	2386
[77]	Strobino (1993)	congenital abnormality, all^1^	Tick bite during pregnancy	1.35^ǂ^	0.72	2.53	1731
[82]	Williams (1995)	congenital abnormality, major ^1^	Tick bite during pregnancy	1.60	0.49	5.23	2386
[77]	Strobino (1993)	congenital abnormality, major^1^	Tick bite during pregnancy	0.59^ǂ^	0.14	2.49	1731
[82]	Williams (1995)	congenital abnormality, minor^1^	Tick bite during pregnancy	1.62	0.64	4.11	2386
[77]	Strobino (1993)	congenital abnormality, minor^1^	Tick bite during pregnancy	1.76^ǂ^	0.90	3.46	1731
[81]	Strobino (1999)	congenital cardiac abnormality	Tick bite during pregnancy	0.93^+^^ǂ^	0.56	1.56	1500
Association with congenital abnormalities and a history (before or during pregnancy) of tick bites, but no LD (GRADE **)							
[77]	Strobino (1993)	congenital abnormality, all^1^	History of a tick bite with no LD^8^	1.46^ǂ^	0.96	2.36	1731
[77]	Strobino (1993)	congenital abnormality, major^1^	History of a tick bite with no LD	1.52^ǂ^	0.75	3.07	1731
[77]	Strobino (1993)	congenital abnormality, minor^1^	History of a tick bite with no LD^8^	1.47^ǂ^	0.87	2.49	1731
Association with congenital abnormalities and mother residing in a LD endemic area compared to a non-endemic LD area (GRADE **)							
[83]	Williams (1988)	congenital abnormality, all^7^	Residence, endemic LD area	0.90	0.49	1.65	421
[82]	Williams (1995)	congenital abnormality, all^1^	Residence, endemic LD area	0.87^ǂ^	0.70	1.06	4814
[82]	Williams (1995)	congenital abnormality, major^1^	Residence, endemic LD area	1.08^ǂ^	0.77	1.53	4814
[82]	Williams (1995)	congenital cardiac abnormality	Residence, endemic LD area	**2.4**^ǂ^	**1.25**	**4.59**	4814
[82]	Williams (1995)	congenital abnormality, minor^1^	Residence, endemic LD area	**0.77**^ǂ^	**0.60**	**0.99**	4814

No est = no estimate is available because there were no events in either group.

*Odds Ratios were calculated from the raw data provided in the paper unless otherwise noted.

^ǂ^ = Odds ratio extracted from the paper.

^+^ = Outcome was adjusted for other variables. [81] is adjusted for maternal age, number of live births, current county of residence, year of birth of study child, occupational x-ray exposure, maternal high blood pressure, and characteristics of residence (wooded area, deer) at the time of birth of the study child. Three studies reported a statistical analysis of the comparability of their exposed and control sampling frames, but did not present adjusted results [77,82,84].

^1^ Congenital abnormalities were summarized in some studies as all abnormalities together and then subdivided into minor abnormalities and major abnormalities.

^2^ Lyme disease serology was conducted in the first trimester.

^3^ Screening for LD positive serology in pregnant women included a single immunoassay.

^4^ Screening test was an immunoassay confirmed by an immunoblot.

^5^ Immunoassay used to screen cord blood

^6^ Immunoblot used to screen cord blood.

^7^ Study only sampled live births, so the impacts of LD that may lead to fetal demise would have been omitted from these results.

^8^ Results represent outcomes for women who had tick bites, but no LD. An association with tick bites is also presented for the same sample including women who had LD and for a subset of births where the physician records were available. The associations reported in the paper were conflicting for minor congenital abnormalities [77].

^9^ The serological test used in this study measured total IgG and IgM.

^10^ Indicates cases in which Lyme disease in the mother was diagnosed following current guidelines [70,71].

The results included six studies where no significant association was reported between adverse birth outcomes (e.g. spontaneous miscarriage and congenital abnormalities) and the mother’s LD status, determined by serology or clinical diagnosis [77–82] (Table 4). The RoB was low (n = 3) and unclear (n = 3) across these six studies, study designs and diagnosis of LD varied, but the conclusions were consistent. This gives this group of studies a *** GRADE indicating some confidence the overall conclusions of this research will not change with future research.

Other exposure measures included seropositive cord blood (n = 3 studies), tick bites during pregnancy (n = 3), history of tick bites (n = 1), and residing in an endemic area (n = 2) (Table 4). No association was shown from cord blood serology (IgG or IgM antibodies) results and adverse birth outcomes [82–84]. In addition, congenital abnormalities overall were not associated with surrogate measures of LD exposure including exposure to ticks or expected exposure to ticks by virtue of living in an endemic area (Table 4) [77,81–83]. Among the results from the LD endemic area of Westchester, New York, USA, a significantly higher odds of cardiac abnormalities and lower odds of minor abnormalities was observed compared to a population in a non-endemic area [82](Table 4). In this study, these associations were shown to be unrelated to a clinical history of LD in the mother and are assumed to be independent of LD [82]. Therefore there was no association between gestational LD or surrogate measures of exposure and adverse birth outcomes across the eight studies in Table 4.

### Risk factors for adverse outcomes in LD exposed populations

**Fig 3:**
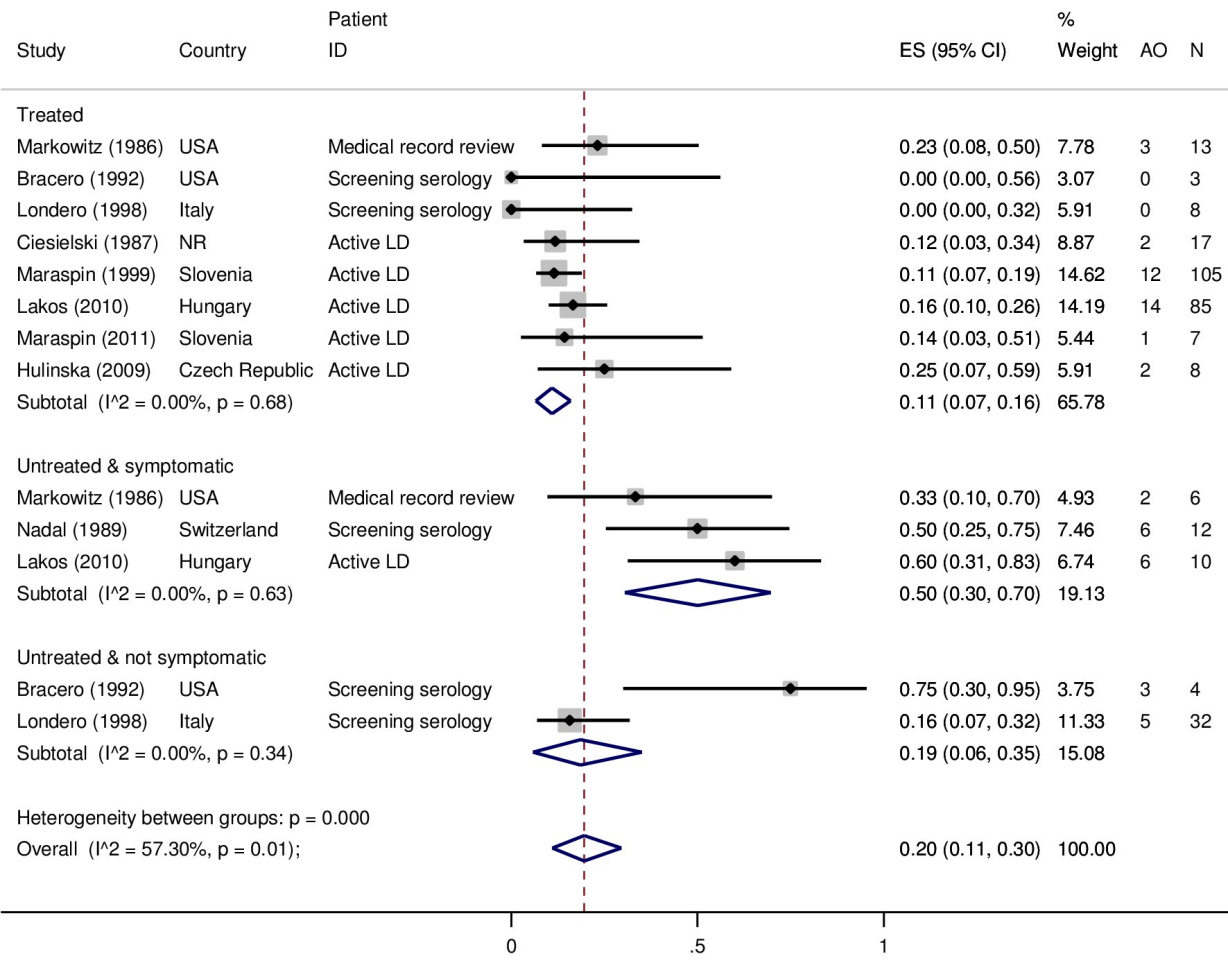
Random effects meta-analysis of nine studies that reported the proportion of women with gestational Lyme disease that experienced an adverse birth outcome. Studies were sub-grouped by treatment status: treated active LD, untreated LD that had a clinical history of LD symptoms, and seropositive with no history of LD. LD status was determined by retrospective medical record review, clinical diagnosis with and without serology or culture, or positive IgG and/or IgM serology. (NR = not reported, AO = Adverse outcome).

There were ten studies that examined various risk factors for adverse birth outcomes among women diagnosed with gestational LD. In nine of these studies there were data on the proportion of adverse birth outcomes in treated and untreated women with gestational LD (Fig 3). LD status was determined in several ways across these studies; active gestational LD diagnosed by a physician (n = 5) [43,44,84–86], retrospective identification based on medical records (n = 1) [44], or a positive serology result on a screening test (IgG and/or IgM) during pregnancy (n = 3) [87,88]. A random effects meta-analysis was conducted to examine the proportion of adverse outcomes across subgroups based on LD and treatment status: i) diagnosed with gestational LD and treated during pregnancy, ii) diagnosed with gestational LD, but not treated during pregnancy and iii) seropositive on a screening test (single immunoassay [80,87] or two tier test [88]) for LD during pregnancy, but did not have a clinical history of illness and consequently was not treated (Fig 3). There was a significantly higher proportion of adverse birth outcomes in the untreated subgroup (50%, 95%CI 30–70, I^2^ = 0%) compared to the group that received treatment (11%, 95%CI 7–16, I^2^ = 0%) (Fig 3). For the subgroup that had a seropositive screening test, but were considered healthy, the results from two studies were quite different from each other and in the meta-analysis this sub-group was not significantly different from the frequency of adverse birth outcomes in the treated and untreated subgroups (19%, 95%CI 6–35, I^2^ = 0%) (Fig 3).

Among these ten studies, two reported that the country level frequency of adverse birth outcomes was the same as the frequency among women in the study. Specifically, treated patients diagnosed with gestational LD had a lower frequency of spontaneous miscarriages and premature births and similar frequency of congenital malformations compared to the country level frequency [43,84]. This suggests that there was no increased risk of adverse birth outcomes among women with treated gestational LD compared to the country birth statistics. The overall GRADE of the studies in Fig 3 is ** meaning that we have limited confidence the results and estimates presented here will not change with future research. This is mainly due to the limited number of studies and observations in each sub-group despite homogeneity across studies and these limitations prevent exploration of potentially important confounding factors such as geographic region, year study was conducted and methods of diagnosis. Therefore, we caution that the summary results are not generalizable beyond the populations studied.

The odds of an adverse birth outcome in gestational LD cases that were treated compared to those that were untreated were described in four of the studies (Table 5), two involving symptomatic women and two involving asymptomatic women. The largest study was from Hungary and reported significantly increased odds of adverse birth outcomes (OR 7.61, 95% CI 1.90–30.51) [84]. The other studies, two from the USA and one from Italy, found no difference between the treated and untreated groups although the results were in the same direction as the study from Hungary [44,80]. Thus, the data suggest there is some evidence that adverse birth outcomes may occur more frequently if gestational LD is not treated.

**Table 5:** Measures of association extracted from six studies examining the odds of an adverse birth outcome in patients with gestational LD by treatment status, timing of exposure, severity and progression of LD. (Significant odds ratios are bolded in the results.).

Ref	Study	Adverse birth outcome definition	Mother’s risk factor	OR*	95% Conf. Interval		N
Association with adverse birth outcomes and untreated compared to treated gestational LD (GRADE **)							
[84]	Lakos (2010)^1^	adverse outcome	Untreated symptomatic LD ^2^	**7.61**	**1.90**	**30.51**	95
[44]	Markowitz (1986)	adverse outcome	Untreated symptomatic l LD ^2^	1.67	0.20	14.05	19
[80]	Bracero (1992)	adverse outcome	Untreated asymptomatic LD	16.33	0.48	555.63	7
[88]	Londero (1998)	adverse outcome	Untreated asymptomatic LD	3.00	0.18	49.32	40
Association with adverse birth outcomes and postnatal treatment of gestational LD compared to treatment during pregnancy (GRADE **)							
[84]	Lakos (2010)	adverse outcome	Postnatal treatment of gestational LD^2^	2.57	0.86	7.69	95
Association with adverse birth outcomes and the length of gestational LD infection (GRADE **)							
[84]	Lakos (2010)	adverse outcome	Clinical gestational LD ^2^	1.00^ǂ^	NR	NR	95
Association with adverse birth outcomes and acquiring LD during the first trimester of pregnancy compared to later in pregnancy (GRADE **)							
[84]	Lakos (2010)	congenital abnormality, all	Clinical gestational LD ^2^	0.17	0.02	1.37	80
[84]	Lakos (2010)	adverse birth outcomes	Clinical gestational LD ^2^	0.92	0.31	2.75	86
Association with adverse birth outcomes and disseminated LD compared to early LD (EM) at diagnosis (GRADE **)							
[44]	Markowitz (1986)	adverse birth outcomes	Disseminated vs. early LD ^2^	3.75	0.45	31.62	19
Association with adverse birth outcomes among EM positive women (early LD) with and without additional LD symptoms (GRADE **)							
[89]	Hercogova (1993)	adverse birth outcomes	Symptomatic gestational LD ^2^	No est.			15

No est = no estimate is available because there were no events in either group.

*Odds Ratios were calculated from the raw data provided in the paper unless otherwise noted.

^ǂ^ = Odds ratio extracted from the paper. One study reported a statistical analysis of the comparability of their exposed and control sampling frames, but did not present adjusted results [77,82,84].

^1^ Results are available for mode (e.g. oral) of antibiotic treatment; all modes are in agreement with the overall result.

^2^ indicates cases in which Lyme disease in the mother was diagnosed following current guidelines [70,71].

Possible risk factors other than LD treatment status were investigated in three studies including trimester of *B*. *burgdorferi* exposure, length of LD during pregnancy, early vs. disseminated gestational LD, and women presenting with an EM only compared to those with an EM and other symptoms of LD [44,84,89](Table 5). None of these studies found a significant association between adverse birth outcomes and these possible risk factors, although most of these studies were small and may have had limited power to detect a difference. For example, in one study with only 19 observations the association was not significant, but there was a higher proportion of adverse birth outcomes in women with disseminated LD (cardiac manifestations and neuroborreliosis; 43% had adverse birth outcomes) compared to early LD (EM only; 17% had adverse birth outcomes) [44]. There were no significant associations between trimester of LD infection and adverse birth outcomes [84] Table 5, or with the frequency of spontaneous miscarriages [77,79]. Birth weight, a surrogate measurement for newborn health, was unrelated to gestational LD in two studies [77,82]. Spirochetes found in the placenta were not associated with adverse birth outcomes in a case series where 3/60 placentas were spirochete positive and all infants were healthy [85]. Coinfection with *Anaplasma phagocytophilum* based on PCR of the blood and/or placenta was reported in one study for 37.5% (3/8) LD positive women that had adverse birth outcomes (n = 2) and healthy twins (n = 1). The small sample size prohibited investigation of the association between coinfection and adverse birth outcomes [90,91].

## Discussion

The literature included in this SR was published between 1985 and 2017, 58% of which are case studies. As evidence, case studies are helpful to generate hypotheses for future research, but cannot be used to further our understanding of a causal relationship, if one exists, between gestational LD and adverse birth outcomes. There were a number of reporting issues in the case studies included in this SR, mostly related to missing or limited information on the mother’s clinical symptoms and the use of diagnostic methods and laboratory tests currently considered unreliable [69–72]. The latter issue also applies to many of the epidemiology studies. This is not to indicate that the results from these studies are false, but that they are questionable, which is a feature of the age of the majority of studies identified for inclusion in this SR.

Diagnosis of LD relies on clinical evaluation, plausible exposure history to infected ticks, and if needed, supplemental diagnostic laboratory tests [70,71]. Reliable test methods would include direct demonstration of *B*. *burgdorferi* in tissues or in culture by IF or PCR using, respectively, specific antibodies or primers, or results of a two tier serological test interpreted by current guidelines, the latter being the most common type of testing for diagnosis of LD [92–94]. Typically, two tier serological testing includes an enzyme immunoassay (EIA) to detect IgM or IgG serum antibodies to *B*. *burdorferi*; positive or equivocal tests are followed by an immunoblot assay (IB, e.g. Western blot) to confirm the positive screening test result [92–94]. Although these guidelines improved the performance of LD testing, all currently available testing options are imperfect [73,95]. Thus, the inadequate sensitivity of serological tests in early LD necessitates physician awareness of LD and careful clinical assessment supported by laboratory testing results when appropriate. Pregnant women who have acquired LD should be treated according to current guidelines [92–94] as the meta-analysis in this SR suggested that treatment of LD during pregnancy was associated with a decrease in the risk of adverse birth outcomes.

The issue of misdiagnosis or misclassification of LD across studies in this SR is important to carefully consider. The use of unreliable tests or test protocols for LD could contribute to misclassification of cases, since 58% of the studies in this SR were conducted prior to the current serological testing guidelines and LD serological tests were used to screen healthy pregnant women in some of the epidemiology studies [77–80,82–84,87,88,92]; there is a measurable risk of false positives and thus misclassification of observations in the sample population [96]. Among several case reports there is little information on the diagnosis of LD and given the age of these articles there appears to be a reasonable risk that some cases were misclassified as gestational LD. Unfortunately, both false positives and false negatives could have occurred in these studies and had an impact on the results in an unknown direction and magnitude possibly resulting in the distortion of detected associations or failure to detect associations, which undermines our confidence in the research results. Considering the potential for misclassification of LD in the included studies, additional research using currently accepted methods of LD diagnosis, an improved understanding of LD, and larger sample sizes (e.g. via large multi-center observational studies) is needed to more adequately explore possible effects of gestational LD and further investigate potential risk factors suggested in this SR.

It is biologically plausible that transplacental transmission of *B*. *burgdorferi* occurs given our understanding of transplacental spirochete transmission for other species of spirochetes (*T*. *pallidum)* in humans [6,7]. There are examples among the 59 case reports included in this SR that suggested transplacental transmission occurs including 4 cases of infection in the fetus or newborn determined using relatively reliable laboratory diagnostic methods. Of these only one case reported clinical LD in the mother, an adverse birth outcome and potential demonstration of *B*. *burgdorferi* in the child; that would provide some confidence that vertical transmission of *B*. *burgdorferi* occurred and may have resulted in a negative outcome for the fetus [57,58]. Examination of the pathological findings from case studies where *B*. *burgdorferi* was identified in various fetal tissues does not provide evidence that the presence of *B*. *burgdorferi* was linked to the pathological findings and there was a lack of inflammatory response noted in several cases [43,44,47,49–52,57,87,89,97]. These findings are in alignment with literature reviews by medical practitioners on this topic [6,98]. Therefore, it is possible that vertical transmission with negative outcomes can occur, but there are knowledge gaps in terms of the pathology and frequency of occurrence.

Common adverse birth outcomes reported across studies in this SR included preterm birth and hyperbilirubinemia, which are also common outcomes in the general population [75,76]. The potential for increased risk of an adverse outcome in women with gestational LD that is not treated was shown in this SR; however an explanation for this was not addressed in the available research and it is very possible that this was due to many factors such as sub-optimal maternal health as opposed to a single specific pathology caused by *B*. *burgdorferi* infection. Congenital malformations of the cardiac or genitourinary system are also among the most common malformations reported [75,99] and were frequently reported in case reports and epidemiological studies in this SR. Hypotheses that there may be higher rates of cardiac malformations as a result of gestational LD were investigated in the early epidemiology studies and case reports included in this SR, but these studies were unable to clarify a relationship with gestational LD [51,52,81]. Given recent research characterising the impact of *B*. *burgdorferi* on the cardiac system, additional work on the teratogenic potential of *B*. *burgdorferi* particularly on the cardiac system may be warranted [100]. However, the evidence in this SR on congenital malformations does not provide sufficient evidence to exclude or confirm a role for *B*. *burgdorferi* in congenital malformations. Future research is needed to address knowledge gaps such as the pathogenesis of *B*. *burgdorferi* infection in the developing fetus and its relationship to adverse birth outcomes.

Several risk factors were investigated based on the pathology observed in the early case reports and our biological understanding of LD. These studies failed to find an association between the mother’s LD status and cardiac, minor or major malformation, spontaneous miscarriages, and fetal death [77,80–82]. Adverse birth outcomes were also not associated with the severity of gestational LD (early vs. disseminated), length of LD during pregnancy, or trimester of infection [44,84,89]. However, there was some evidence of increased risk of adverse outcomes in symptomatic women who were not treated with antibiotics, and it is possible that associations with rare or infrequent outcomes were not detected because the sample sizes (or number of LD cases) in most of the epidemiological studies was small and the range of reported outcomes was quite large.

There are several limitations to the evidence included in this SR. This includes limited generalisability of the results to populations other than those studied as there is not enough research to determine whether population differences exist and how they could have impacted the findings. Country level or regional rates for adverse birth outcomes are influenced by many factors related to socio economic factors, healthcare, and genetic predispositions that should be considered when weighing the generalizability of the data [77,82]. Other possible sources of variation in the frequency and type of outcomes include differences among genospecies of *B*. *burgdorferi* (e.g. *B*. *burgdorferi sensu stricto*, *B*. *afzelii*, *and B*. *garinii*) that can cause different manifestations of LD. The data on *Borrelia* species was scarce among the studies in this SR and should be considered in the design of future research to clarify if there are different outcomes or impacts of gestational LD depending on the pathogen [6].

## Conclusion

This SR summarizes the research and anecdotal evidence on the potential impact of gestational LD on adverse birth outcomes. Overall there is a limited amount of evidence; with 29 case report articles and 17 epidemiological studies on this topic, and the results highlight a number of knowledge gaps and significant uncertainty about the impact of LD during pregnancy. Due to the variability in the study size and study design, the lack of evidence in the epidemiological research does not rule out uncommon consequences of LD during pregnancy. There is some evidence to suggest that it is biologically plausible for *B*. *burgdorferi* to be vertically transmitted to the fetus, however these studies have been unable to define a characteristic pathological effect of *B*. *burgdorferi* infection in the fetus, thus there are significant knowledge gaps about the relationship of *B*. *burgdorferi* infection and adverse birth outcomes [32]. Given the uncertainty around the impact of *B*. *burgdorferi* on the fetus and the consistent evidence suggesting fewer adverse birth outcomes if LD is promptly treated, it is recommended that physicians continue to remain thorough in their diagnosis and treatment of LD in pregnant women and that new research address the knowledge gaps identified in this review.

## Supporting information

S1 Text Systematic review protocol. (PDF)

S2 Text List of included studies. (PDF)

S1 Table PRISMA checklist. (DOC)

S2 Table dataset. (XLSX)
