# Supplementary material for: A systematic review on the impact of gestational Lyme disease in humans on the fetus and newborn
Source: PLoS One. 2018 Nov 12;13(11):e0207067. doi: 10.1371/journal.pone.0207067 (PMC6231644; doi:10.1371/journal.pone.0207067)
Supplement: S1 Text — (PDF) [file pone.0207067.s001.pdf]

Systematic Review Protocol

What is the evidence that gestational Lyme disease in humans causes adverse birth outcomes including congenital abnormalities?

Authors:

Lisa Waddell[1], Judy Greig[1]

[1]Public Health Risk Sciences Division of the National Microbiology Laboratory, Public Health Agency of Canada.

Contact: Lisa Waddell, Tel:226-979-7174 or email: lisa.waddell@phac-aspc.gc.ca

Important Dates:

Evidence published up to October 16, 2017

Table of Contents

Systematic Review Protocol 1
Authors: 1
Important Dates: 1
Objectives of the Systematic Review 3
  Study Question 3
Methods 3
  Review Team Expertise and Responsibilities 3
  Search Strategy 3
    Algorithms 3
    Databases 3
    Search Verification 4
    Grey Literature Search 4
    Search Results and Database Specific Search Details: 4
  Relevance Screening (RS) 6
    Inclusion / Exclusion criteria 6
  Risk-of-bias assessment and GRADE: 6

Data Extraction 7

Review management: 7

Data analysis: 7

Appendix 8

Relevance screening Title/Abstract tool 8

Quality Assessment and Data Extraction tools 8

Objectives of the Systematic Review

Conduct a systematic review to identify, critically appraise, extract data on relevant outcomes and synthesize the literature on the impact of Lyme disease during pregnancy. Methods following the best practices for synthesis research prescribed by the Cochrane Collaboration will be utilized to undertake this project.

Study Question

What is the evidence that gestational Lyme disease in humans causes adverse birth outcomes including congenital abnormalities?

Inclusion criteria: is all evidence examining the impact of maternal Lyme disease in humans for any outcome conducted anywhere in the world.

Exclusion criteria: research on non-human hosts including animal models of the impact of *Borrelia burgdorferi* infection on pregnancy, fetal and newborn outcomes.

Methods

Review Team Expertise and Responsibilities

Member	Organization	Project Role*
Lisa Waddell	RISK - Guelph	Synthesis expertise- co-lead
Judy Greig	RISK - Guelph	Synthesis expertise – co-lead
Nicholas Ogden	NML	Expert Advisory
Robbin Lindsay	NML	Expert Advisory
Allison Hinckley	CDC	Expert Advisory

Search Strategy

The search algorithm below will be executed in 3 bibliographic databases Pubmed, Scopus and Embase on October 16, 2017 via the Public Health Agency of Canada library. A search verification strategy was employed to identify any literature that was omitted from the bibliographic database search. Search results will be downloaded, deduplicated and managed in reference management software, Endnote.

Algorithms

((lyme or borrelia or borreliosis) and (pregnancy or pregnant or maternal or fetus or foetus or newborn or congenital))

Databases

Pubmed, Scopus and Embase

Search Verification

Reference lists of a minimum of four relevant publications (book chapters, literature reviews and/or primary research articles) will be scanned for relevant citations missed by the electronic search. If we are still finding missed publications after four papers, additional papers will be selected and the reference lists will be scanned until we no longer identify potentially relevant research that has not been captured already.

Grey Literature Search

The following websites were searched by using simple combinations of the keywords lyme or borrelia and pregnancy or fetus or newborn to identify potentially relevant pages and each page was screened for primary data related to the topic. Potentially relevant grey literature would be added to the citation list for relevance screening. However, no additional citations were found.

1. Center for disease control and prevention (CDC) https://www.cdc.gov/
2. European Center for Disease Control and Prevention (ECDC) https://ecdc.europa.eu
3. Public Health Agency of Canada, https://www.canada.ca

Search Results and Database Specific Search Details:

Database search results for Pubmed, Scopus and Embase October 16, 2017.

Pubmed: n=392 (no limits, mapping on)
(((lyme or borrelia or borreliosis) and (pregnancy or pregnant or maternal or fetus or foetus or newborn or congenital)))

Scopus: n=403, limited to articles, conference proceedings and journal articles (excluded books, reviews, patents etc.)
TITLE-ABS-KEY ((((lyme OR borrelia OR borreliosis) AND (pregnancy OR pregnant OR maternal OR fetus OR foetus OR newborn OR congenital)))) AND (LIMIT-TO (DOCTYPE , "ar") OR LIMIT-TO (DOCTYPE , "cp")) AND (LIMIT-TO (SRCTYPE , "j"))

Embase: n=534 limited to research or work about humans using Embase' filter.

((lyme or borrelia or borreliosis) and (pregnancy or pregnant or maternal or fetus or foetus or newborn or congenital)).mp. [mp=title, abstract, heading word, drug trade name, original title, device manufacturer, drug manufacturer, device trade name, keyword, floating subheading word] –**limit to human**

Search Verification: Reference lists of the following publications were evaluated for relevant citations missed by the electronic search (Walsh *et al.*, 2007), (Mylonas, 2011), (Gardner, 1995), (McClure & Goldenberg, 2009, Gardner, 2001, Shapiro, 2011)

Table: List of 13 citations identified by search verification

Reference	Fate in Review
Ciesielski CA, Russell H, Johnson S, et al.: Prospective study of pregnancy outcome in women with Lyme disease (abstract). 27th ICAAC, 1987.	Included
Lavoie PE, Lattner BP, Duray PH, Malawista SE, Barbour AG, Johnson RC. 1987. Culture positive, seronegative transplacental Lyme borreliosis infant mortality. Arthritis Rheum 3(suppl):S50.	Included
Dlesk A, Broste SK, Harkins PG, McCarty PA, Mitchell PD. 1989. Lyme seropositivity and pregnancy outcome in the absence of symptoms of Lyme disease. Arthritis Rheum 32(suppl):S46	Included
Trevisan G, Stinco G, Cinco M. Neonatal skin lesions due to a spirochetal infection: a case of congenital Lyme borreliosis? Int J Dermatol 1997;36:677–680.	Included
Sigal LH. Pregnancy complicated by Lyme disease. June 2005. Available athttp://www.uptodate.com. Accessed June 2006.	Can't obtain – may be an editorial on a subscription site.
Podolsky ML. Lyme disease in pregnancy: the new great imitator. Clin Adv Treat Infect. 5(5):1, 1991 (probably a review)	Can’t obtain – likely a review
Lampert F. Infantile multisystem inflammatory disease: another case of a new syndrome. Eur J Pediatr 144:593, 1986	Included
Williams CL, Benach JL, Curran AS et al. Lyme disease during pregnancy: a cord blood serosurvey. Ann N Y Acad Sci 539:504, 1988	Included
Hercogova J. Moidlova M, Zirny J et al. Could borrelia found in the placenta influence the fetus? Study of 19 women with erythema migrans during pregnancy. In Program and Abstracts of the 6th Interantioanl Conference on Lyme Borreliosis. Bologna, Italy, Societa Editrice Esculapio, 1994, p76 (abstract No PO 06T)	Can’t obtain, have a full paper that precedes, Hercogova 1993, this meeting with similar information (n=15).
Bracero LA. Wormser GP, Leikin E, Tejani N, Prevalence of seropositivity to the Lyme disease spirochete during pregnancy in an epidemic area. A preliminary report. J Matern Fetal Invest. 2:265-268, 1992	Included
Duray PH, Steere AC. Clinical pathologic correlations of Lyme disease by stage. Ann N Y Acad Sci 539: 65, 1988.	Not relevant
Preiur HM, Giscelli C. Arthropathy with rash, chronic meningitis, eye lesions and mental retardation. J pediatr 99: 79, 1981.	Not relevant
Robinson TT, Herman L, Birrer RB, Lyme carditis; a rare presentation in an unexpected setting. Am J emerg Med 16(3): 265-269, 1998.	Not relevant

Grey literature search: No additional citations were found.

Total deduplicated citations to be screened for relevance= 753

Relevance Screening (RS)

The relevance screening level will be done on the title, abstract and keywords where available. There is 1 question and the answers are based upon the inclusion / exclusion criteria and can be found in the appendix.

Inclusion / Exclusion criteria

Potential inclusion/exclusion criteria

1) Time frame – no time frame
2) Country – All
3) Language – English, French. All other languages will be identified, the paper will be obtained and we will evaluate if there are resources to include papers in other languages.
4) Document Type: Any article, report or thesis containing primary data (data collected by the author/ author's organisation). All literature reviews, letters, commentaries, new reports etc that do not contain primary data will be excluded at relevance screening (based on title/abstract) or the beginning of the second level (based on the full paper).
5) Study design – all
6) Population- studies on humans and the impact of infection on human pregnancies. Animal models and other studies on *B. burgdorferi* infection in animals will be identified as such at relevance screening and excluded from the review.
7) Pathogen – Any of the *B. burgdorferi* group of borrelia.

Risk-of-bias assessment and GRADE:

Relevant studies that meet all eligibility criteria will undergo a risk of bias assessment. Most relevant studies are expected to be case reports, cross-sectional or cohort design. A risk of bias assessment tool will be developed to assess the internal validity of the study, ie: whether it answers the research question correctly. In this sense we are assessing systematic error, deviation from the truth, in results or inferences (Balshem *et al.*, 2011, Guyatt *et al.*, 2011b, Higgins & Altman, 2008). These biases may vary in direction and magnitude; however it is impossible to know the extent that the biases have influenced the results of a study (Higgins & Altman, 2008). The risk of bias evaluates selection bias, performance bias, attrition bias, detection bias, reporting bias and confounding bias. The results of this help us to use the GRADE criteria to grade the evidence (Balshem et al., 2011, Higgins & Green, 2011, The, 2013).

For each outcome a Grading of Recommendations Assessment, Development and Evaluation (GRADE) criteria was applied (Guyatt *et al.*, 2011a, Higgins & Green, 2011, The, 2013). The risk of bias assessment aims to assess the internal validity of the study which informs one of the GRADE criteria (Higgins & Green, 2011, Higgins & Altman, 2008). The other 6 GRADE components include indirectness of

evidence, unexplained heterogeneity, imprecision/high prevision of results, high probability of publication bias, studies are underestimating the measure of effect and detection of a dose-response gradient. GRADE criteria are summarized across groups of like studies to indicate the level of confidence in the current evidence (The, 2013). The one to four star grading system indicates: **** high confidence that the effect estimate is close to the true effect; *** moderate confidence in the effect estimate, but future studies may be substantially different; ** limited confidence in the estimate of effect, the true effect may be substantially different; * very little confidence in the estimate of effect, the true effect is likely to be substantially different (Balshem et al., 2011, Guyatt et al., 2011b, Schunemann *et al.*, 2011).

Data Extraction

A data extraction form was developed to extract general study information and capture specific quantitative and descriptive outcomes from each study captured in this review. See the Data extraction form in the Appendix.

Review management:

To ensure rigour in the review process, all steps will be conducted using pre-tested tools by two independent reviewers. All references identified in the review will be de-duplicated in the reference management program Endnote© (Thomson Reuters, USA) and imported into the systematic review management software DistillerSR (Evidence Partners, Ottawa, Canada) to facilitate review management and progress. All extracted data will be downloaded as Excel spreadsheets for analysis.

Data Analysis:

Extracted data from relevant articles will be descriptively characterized and summarized. Quantitiatve outcomes including prevalence, counts, and measures of association will be converted where necessary into a standard effect size metric, based on the mostly commonly used measure reported in relevant studies (Borenstein *et al.*, 2009). Data will then be stratified into sufficiently comparable subgroups if there are some, and random-effects meta-analysis will be conducted to determine average effect sizes and the extent of heterogeneity across studies in each subgroup [8]. Meta-analysis will be conducted using Stata (StataCorp LP, College Station, USA). We do not anticipate having outcomes with enough data to warrant meta-analysis.

Appendix

Relevance screening Title/Abstract tool

Is the citation primary research on pregnant women, fetus or newborns and the impact of *Borrelia burgdorferi* (Lyme disease) infection during any stage of pregnancy?

- ☐ Yes – primary research on humans
- ☐ Yes – relevant literature **review or guidelines**
- ☐ No – paper is about **treatment** of LD during pregnancy in humans
- ☐ No – **animal** model/study about the impact of *B. burgdorferi* infection during pregnancy.
- ☐ No - not relevant

Quality Assessment and Data Extraction tools

**Relevance Confirmation with full paper**		
**Question**	**Options**	**Comments**
**1) Is the citation primary research on pregnant women, fetus or newborns and the impact of *Borrelia burgdorferi* (Lyme disease) infection during any stage of pregnancy?**	☐ Yes – primary research on humans<br>☐ Yes – relevant literature **review or guidelines**<br>☐ No – paper is about **treatment** of LD during pregnancy in humans<br>☐ No – **animal** model/study about the impact of *B. burgdorferi* infection during pregnancy.<br>☐ No - not relevant	**Lyme disease** is caused by the bacterium *Borrelia* spp. and is transmitted to humans by tick vectors. **Primary research**: a study where the authors collected and analyzed their own data – may use quantitative or qualitative methods or both to investigate the research question and report original results.
**2) What language is the article published in?**	☐ English<br>☐ French<br>☐ Other: ___specify	
**3) What *Borrelia burgdorferi* sensu lato was the cause of the infection(s) described in the paper?**	☐ BB s.l. (specify): _______________<br>☐ Not relevant Borrelia.	* Indicate (NS) if the type of *Borrelia* was not specified (due to only serological results, failure to isolate, not reported etc.)
**4) What continent and country are the samples from?**	☐ North America<br>☐ Europe<br>☐ Asia	

	☐ South America<br>☐ Middle east<br>☐ Australia<br>Country (specify): ________	
**5) Indicate whether maternal, fetal or newborn outcomes are reported in the paper.**	☐ Maternal outcomes<br>☐ Miscarriage/ pregnancy loss<br>☐ Fetal outcomes (Outcome measured before birth)<br>☐ Newborn outcomes (Outcome measured after birth)<br>☐ Long-term impact of congenital defects (e.g. autism)<br>☐ Other potentially relevant outcome: specify _____<br>☐ No relevant outcomes reported	
* If an exclusion criteria was selected for any of the above questions, please submit the form and do not proceed to Risk of Bias assessment.		
**Risk of bias assessment**		
6) What is the publication year of this article?	☐ [text]	Enter year or NR if not reported
7) In what year were the samples collected?	☐ [text]	Enter year or NR if not reported
8) What is the study design?<br><br>*(Check all that apply)*	☐ Case Report/Case series<br>☐ Cross-sectional<br>☐ Cohort<br>☐ Case-control<br>☐ Other: [text]	**Report ONLY study design(s) relevant to the research question.**<br><br>**Observational study:** Assignment of subjects into an exposed group versus a control group is outside the control of the investigator.<br><br>• **Cross-sectional:** Examines the relationship of a risk factor and outcome (disease) at a point in time on representative samples of the target population.<br>• **Cohort study**: is a study in which

		individuals with differing exposures to a suspected risk factor are observed through time for occurrence of an outcome<br>• **Case-control study**: compares exposure to the risk factor in subjects who have an outcome (the 'cases') with subjects who do not have the outcome, but are otherwise similar (the 'controls') and drawn from the same sampling frame. **There may be an occasional experimental design** – please include under "other"
9) **Was the allocation sequence adequately generated? (GRADE 1-1)**<br>"RCT, ChT Selection bias: systematic differences between baseline characteristics of the groups that are compared."	☐ Yes (low risk of bias): [text]<br>☐ Unclear: [text]<br>☐ No: [text]<br>☐ NA – not an experiment [RCT, ChT]	**Yes:** allocation sequence is described in sufficient detail ___page #__.<br>**Unclear:** they simply stated that it was "randomized" (formerly partial).<br>**No:** Sample drawn without a formal<br>process of random selection: judgment, convenience, purposive.
10) Was the allocation sequence adequately concealed from the participants and the researcher? (GRADE 1-2)<br>"RCT, ChT Selection bias: systematic differences between baseline characteristics of the groups that are compared."	☐ Yes: [text]<br>☐ Unclear: [text]<br>☐ No: [text]<br>☐ NA – not an experiment [RCT, ChT]	Yes: concealment was sufficient and allocation was unlikely to be foreseen (in advance of or during enrollment) ___ page #___<br>Unclear: author only indicated "blinding" or "concealed treatment" was used.<br>No: no concealment strategy described or was insufficient.
11) **Was the level of exposure representative of exposure in the population of interest?**	☐ Yes: [text]<br>☐ Unclear: [text]	Yes: Does the sample reflect the proportion of high risk and low risk people in the population the

**(GRADE 1-3)**<br><br>"Cohort Selection bias: systematic differences between sample and target population."	☐ No: [text]<br><br>☐ NA- not a cohort study	investigator would like to extrapolate the results to?<br><br>No
12) Were the study participants (samples) selected randomly so the sample reflects disease and exposure in the population of interest? (Cross-sectional)<br><br>OR<br><br>Were the controls selected from the same source population as the cases? (case control)(GRADE 1-4)<br><br>"Selection bias: systematic differences between sample and target population or for case control studies between the groups being compared and an appropriate range of clinical severity."	☐ Yes<br><br>☐ Unclear- too few details are available to make a clear judgement<br><br>☐ No: [text]<br><br>☐ NA- not a case control, cross-sectional	Selection bias:<br><br>Yes: Random selection of the study participants or samples are stated and described or objective identification of controls in case control stated.<br><br>No: Study participants were selected non-randomly or were not Described
13) Was blinding for patients/sample and individuals involved in the care of the patients/sample appropriate? (Patient, doctor, vet, health care worker) *Please note if there is a different answer for different outcomes.* ***(GRADE, 1-10)***<br><br>"All studies: Performance bias: Systematic differences between groups in the care that is provided, or in exposure to factors other than the intervention of interest."	☐ Yes<br><br>☐ Unclear, reported that blinding was used [text]<br><br>☐ No: [text]<br><br>☐ NA case report	Was knowledge of the status of the individual or sample adequately prevented during the study?

14) Was blinding for the outcome assessor, statistician and manuscript writer appropriate? *Please note if there is a different answer for different outcomes.* ***(GRADE, 1-11)***<br><br>"All studies: Detection bias: Systematic differences between groups in how outcomes are determined."	☐ Yes (low risk of bias)<br><br>☐ Unclear, reported that blinding was used [text]<br><br>☐ No: [text]<br><br>☐ NA case report	Was knowledge of the status of the individual or sample adequately prevented during the study?
15) **Incomplete outcome data;** Was loss to follow-up equal in both groups ? (GRADE 1-5)<br><br>"experiments, cohort, long prev: Attrition bias; Systematic differences between groups in withdrawal from the study."	☐ Yes (low risk of bias)<br><br>☐ Unclear: [text]<br><br>☐ No: [text]<br><br>☐ NA case report	Describe the completeness of outcome data for each main outcome, including attrition and exclusions from the analysis.<br><br>Unclear: there are too few details to make a judgment.<br><br>No: explain i.e. there was loss to follow-up and it was not clearly reported, appears to be high>20%, thus there is concern
16) **Incomplete outcome data:** If observations were excluded from the analysis, were the exclusions appropriate and/or clearly justified in the text?<br><br>(GRADE 1-6, new)<br><br>"all studies: Reporting bias: Systematic differences between reported and unreported findings"	☐ Yes: (low risk of bias)<br><br>☐ Unclear: [text]<br><br>☐ No [text]<br><br>☐ NA case report	Yes: [text]<br><br>Unclear: there are too few details to make a judgment.
17) Does the study appear to have reported all intended outcomes? (GRADE 1-7, new)<br><br>"Reporting bias: Systematic differences between reported and unreported findings (e.g.	☐ Yes (low risk of bias)<br><br>☐ Unclear: [text]<br><br>☐ No: [text]	Reporting bias<br><br>Yes: page#___<br><br>Unclear: too few details are available to make a clear judgement

only statistically significant findings reported)”		No: explain_
18) Have confounders been appropriately identified and accounted for? (GRADE 1-9)<br><br>“Confounding bias: a variable that distorts the relationship between the exposure and outcome of interest. Particularly an issue in observational studies.”	☐ Yes (low risk of bias)<br><br>☐ Partial: [text]<br><br>☐ Raw Data<br><br>☐ No: [text]<br><br>☐ NA case report	Confounding bias:<br><br>Yes: All-important confounding factors were identified, accounted for by exclusion, matching or analysis. (sex, age, ethnicity etc.)<br><br>Partial: some confounders controlled but not all of them.<br><br>Raw data so post hoc analysis could be done<br><br>No: Not stated.
19) Was the study free of other problems that could put it at a high risk of bias? (GRADE 1-8, new)<br><br>“Other performance bias, detection biases, non-response bias, recruitment bias, misclassification, or biases related to poor study design and conduct.” e.g.<br><br>- Observational studies, **non-response bias** means that only a particular subset participated and is not reflective of the general population.<br>- Statistical analysis was not appropriate, the results are invalid and there is insufficient raw data.<br>Misclassification bias: Were the methods to classify samples into exposure, disease and outcome groups standard and reliable?	☐ Yes (low risk of bias)<br><br>☐ Unclear: [text]<br><br>☐ No: [text]	All other bias' that could put the study at risk.<br><br>e.g.: non-randomization, clusters, stopping the study early without explanation, sample size intended (these are NOT more likely to have biased results)<br><br>Vs.<br><br>Obvious imbalance in baseline factors that have an influence on the outcome. Outcome assessment can become biased. Selective reporting of subgroups can be biased (these ARE more likely to have biased results)<br><br>Yes, I have no additional concerns about the design and/or conduct and reporting of this study.<br><br>No, the following are concerns I have that this study is at risk of bias. (list with page#)

**20) Based on the risk of bias questions *(GRADE 1-1 to 1-11)* please indicate the <u>overall</u> risk of bias for this study (GRADE 1-12, new)**	☐ **Low RoB**<br><br>☐ **Unclear RoB**<br><br>☐ **High RoB**	Low risk of bias: no biases were indicated in the assessment. Thus plausible bias is unlikely in all key domains (within this study). (Across studies: most studies indicate low risk)<br><br>Unclear risk of bias, there are plausible bias that raises doubt about the results as some key domains are "unclear (within this study). (Across studies: most information is from low or unclear RoB).<br><br>High Risk of bias indicates that in one or more of the domains serious plausible bias was identified (within the study). (Across studies: The proportion of studies that are at high risk of bias is sufficient to affect the interpretation of results.)
**21) Does this study examine the question of interest directly?** (GRADE 2-1, new)	☐ Yes, this study directly addresses the question of interest. Please state which outcomes were directly answered [text]<br><br>☐ No, this study indirectly examines the question of interest. Please state which outcomes were not directly answered [text]	A study may indirectly address the question of interest if:<br><br>e.g. risk factors we wish to compare are measured independently in two separate trials compared to controls.<br><br>e.g. the population, risk factors , comparisons or outcomes were not exactly what we are trying to draw conclusions for.<br><br>* Downgrading occurs if there is reason to believe that there may be differences in the conclusions due to indirectness.
22) Was this study funded by or was there involvement of individuals employed by or affiliated with industry (drug or chemical) or a special interest / advocacy group? (GRADE 5-1,	☐ No, There are no concerns based on the authors, funding and declarations in the paper.<br><br>☐ Yes[text]	This criteria for down-grading would be used if all or most of the trials captured are industry funded or declare heavy sponsor involvement (e.g. advocacy groups), in which case there are concerns that studies of null or negative effect may have been suppressed from

new)<br><br>Identify in text box details:<br><br>-if there was a declaration of involvement.<br><br>- if the study was funded by such an organization<br><br>-if the author's affiliation was for such an organization.		publication.<br><br>**Select yes and provide details if there was industry or advocacy group sponsorship.**
23) Is there reason to believe that due to the population studied, the magnitude of effect (association) of the risk factor (outcome) may be underestimated? (GRADE 6-1, new)	☐ Yes, an underestimation is likely<br><br>☐ No, there is no reason to believe the estimated effect is underestimated.	You would answer yes ONLY if there was good reason to think that the study underestimated the potential association or effect of a risk factor due to the population that was sampled.<br><br>e.g. The magnitude of association was lower than it likely is in the general population because the comparison group has a similar disease which in also more likely to result in having the exposure of interest.
24) Was a dose-response gradient detected for the exposure being examined? (GRADE 7-1, new)	☐ Yes, dose-response gradient detected. Please state which outcomes demonstrated a dose-response gradient [text]<br><br>☐ No: no does-response gradient reported. Please state which outcomes did not demonstrated a dose-response gradient [text]	If a dose response gradient is demonstrated in some or all of the studies, this increases our confidence in the findings of the study and thus we can consider upgrading the evidence.

Data Collection Forms:

1. Case report information (1 form per case)
2. Epidemiological information (summary data on case series, cross-sectional, case control and cohorts): prevalence or association data (1 outcome per form)

Note: if testing methods are referenced to another paper and sufficiently described

please note the reference.		
**Data extraction Case report form**<br>(1 form per pregnancy case, multiple infants okay.)		
Pregnant Mother data:	☐ Date: [text]<br>☐ Place: [text]<br>☐ Age: [text]<br>☐ Other demographic information: [text]<br>☐ Number of weeks gestation at time of miscarriage or birth: [text]<br>☐ When did they acquire Lyme disease relative to pregnancy? [text]<br>☐ What clinical symptoms of Lyme disease were described, note duration and if they persisted to the end of pregnancy? [text]<br>☐ What tests were done to confirm Lyme disease and when were they conducted?[text]<br>☐ Was their Lyme disease treated? Record when, length and what was used for treatment: [text]<br>☐ Did the mother have other sequelae or co-infections? [text]<br>☐ Other descriptors of the mother that should be noted? [text]	Date: year is fine<br>Tests: be specific listing each test, at what time in pregnancy/after pregnancy and note if test is specific for *B. burgdorferi* in test description
Placenta outcomes	☐ The placenta was tested, describe testing [text]	

	☐ Was the placenta positive? Describe results [text]<br>☐ Other descriptors of the placenta that should be noted [text]	
Cord blood outcomes	☐ Describe the test methods for cord blood results. [text]<br>☐ Describe the results of cord blood testing [text]<br>☐ Other descriptors of cord blood outcomes that should be noted? [text]	
Fetal outcomes	☐ When (stage of pregnancy) did miscarriage or fetal death occur? [text]<br>☐ Results of autopsy [text]<br>☐ Describe tests conducted and whether it was specific for *B. burgdorferi. [text]*<br>☐ Results of testing fetus for BB.<br>☐ Other important descriptors of fetus outcomes that should be noted [text]	e.g. Still born at 35 weeks.<br>e.g. No external malformations, atrioventricular canal ventricular septal defect.<br>e.g. indirect immunofluorescence (not further described) in a retrospective examination of fetal autopsy tissue. Spirochetes identified in "tissue" (not further specified)
Newborn/Infant outcomes	☐ What pregnancy week was the child born? [text]<br>☐ Sex of child: [text]<br>☐ Describe health at birth: [text]	e.g 39th week<br>e.g. male/ female<br>e.g. died 30 minutes after birth, jaundice, Infant Developed respiratory distress within first day of life,

	☐ Describe any symptoms of Lyme disease infection in the newborn (note time of appearance) [text]<br>☐ Describe the tests conducted to establish Lyme disease in the child (note whether the test was specific to *B. burgdorferi*) [text]<br>☐ Describe the results to establish Lyme disease in the child [text]<br>☐ Describe how the child was treated and if treatment was successful. [text]<br>☐ If the child died, describe the physical findings of the autopsy: [text]<br>☐ If the child died, describe the post-mortem testing for Lyme disease or spirochetes: [text]<br>☐ What were the results of the post-mortem testing for Lyme disease or spirochetes:<br>☐ Other important descriptors for the child/new born outcomes? [text]	hypoglycemia and fever. Infant was healthy after treatment. etc.<br>e.g. 3 weeks old to 9 months old he had relapsing multiple annular erythema.<br>e.g. Culture (no description), No pathogens detected.<br>e.g. large (1 cm diameter) ventriculoseptal defect and showed an absence of the left hemidiaphragm with herniation of abdominal viscera into the left hemithorax.<br>e.g. Culture tissues= modified Kelly's medium and dark field microscopy: Spirochetes cultured from liver. Indirect Immunofluorescence (monoclonal antibody H5332): immunoflouresced in myocardium, adrenal, brain. Warthin-Starry silver stain: identified spriochetes in the myocardium, liver and brain. Serology was negative (test not described).
Additional comments	[text]	

Data Extraction of Epidemiological information

Appropriate for outcomes that summarize data on case series, cross-sectional, case control and cohorts. Extract 1 outcome per form, multiple forms per study possible. Information can include count information,

prevalence data or association data.		
Describe the sampling frame *(you only need to do this 1x per study unless the data varies, I can copy and paste through the dataset ;)*	Description of sampling frame [text]<br><br>Location [text]<br><br>Place [text]<br><br>Date [text]	e.g. state, county, city<br><br>e.g. name of facility<br><br>e.g. sampling dates<br><br>e.g. Prospectively enrolled consecutive asymptomatic LD positive or equivocal ELISA pregnant women.
Describe the **exposure** reported in this form include sample & outcome options to determine +ve/-ve:	[text]	This should be the establishment of Lyme disease in the mother +ve vs. -ve, spirochetes in the placenta or cord blood, may be treated vs. untreated LD in pregnancy etc in the sample.
Describe the test conducted to assess the **exposure** (e.g. clinical assessment, Lyme disease testing etc.)	[text]	
Describe the **outcome** reported in this form sample & outcome options to determine +ve/-ve:	[text]	This should be the health of the newborn vs. abnormalities, # of miscarriages vs. full term pregnancies, etc. (could also be rate in case series compared to a national rate of negative pregnancy outcomes)
Describe the test conducted to assess the **outcome** (e.g. clinical assessment, Lyme disease testing etc.)	[text]	
**Dichotomous/Ordinal Data**<br><br>(Note; if prevalence is the outcome, just fill in the data for group 1.)	☐ Define group 1 [text]<br>☐ Define group 2 [text]<br>☐ Specify "positive" [text]<br>☐ Specify "negative" [text]<br>☐ No. positive in group 1 [text]<br>☐ No. negative in group 1[text]<br>☐ Proportion positive in	**Only answer based on how outcome data are REPORTED**<br><br>**Dichotomous:** Sufficient information includes:<br><br>• Numerator ***and*** denominator, ***or***<br>• proportion + EITHER numerator or denominator ***or***

	group 1 [text]<br>☐ N in group 1 – *if 2x2 is not provided* [text]<br>☐ No. positive in group 2 [text]<br>☐ No. negative in group 2[text]<br>☐ Proportion positive in group 2 [text]<br>☐ N in group 2 – *if 2x2 is not provided* [text]<br>☐ If greater than two groups, specify data for other groups [text]<br>☐ Specify type of measure of association reported (OR, RR, etc.) [text]<br>☐ Measure of association value and measure of variability as reported [text]<br>☐ Was measure of effect adjusted for other variables? Please specify: [text]<br>☐ Define what the measure of effect means [text]	• Measure of association (e.g. odds ratio, relative risk) + EITHER a measure of variability (SE, CIs, variance) *or* an exact P-value<br><br>e.g. Odds Ratio<br><br>e.g. OR 2.5 (2.1-2.9), OR 2.5 (SE 0.4) etc.<br><br>If the measure of effect is different across confounders, please specify these results as well.<br><br>e.g. The odds of detecting abnormalities in newborns were 2.5 times higher in LD seropositive pregnant women.
Continuous outcome?<br><br>(hidden unless selected)	☐ Yes *(expand below)*	
**Raw continuous data (group 1 vs. group 2 data):** Raw continuous data in each group (final outcome measure)	☐ Define group 1 [text]<br><br>☐ outcome in group 1 [text]<br><br>☐ SD in group 1 [text]<br><br>☐ N in group 1 [text]<br><br>☐ Define group 2 [text]<br><br>☐ outcome in group 2 [text]<br><br>☐ SD in group 2 [text]	**Continuous:** Sufficient information includes:<br><br>• Mean, sample size, + EITHER a measure of variability (e.g. SD, CIs) *or* exact P-value/t-value ***or***<br>• Sample size and P-value/t-value from t-test ***or***<br>• Difference in means and a measure of variability (SD, SE, CIs, variance) ***or***<br>• Difference in means, sample size, + EITHER a common SD *or* an exact P-

	N in group 2 [text]<br><br>P-value (exact only) [text]<br><br>T value [text]<br><br>For matched studies, specify pre/post correlation [text]<br><br>Outcome units [text]<br><br>Outcome scales (i.e. lowest/highest possible values and if higher values are a more desired outcome)<br><br>Detection limit or analytical sensitivity of test [text]<br><br>If greater than two groups, specify data for other groups [text]	value /t-value<br><br>(e.g. higher behaviour/knowledge scores) or less desired (higher Borrelia counts)
**Difference in means (between exposed/control groups)**	Define the two groups being compared [text]<br><br>Difference in means (value) [text]<br><br>N (total sample size) [text]<br><br>Common SD [text]<br><br>SE [text]<br><br>Variance [text]<br><br>95% CI [text]<br><br>P value (exact only) [text]<br><br>T value [text]	

	Outcome units [text]<br><br>Define the interpretation of the summary measure [text]<br><br>Outcome scales (i.e. lowest/highest possible values and if higher values are a more desired outcome)<br><br>Detection limit or analytical sensitivity [text]<br><br>Was outcome adjusted for other variables? Please specify: [text]	
Other outcomes	[text]	e.g. Pearson correlations
Additional comments:	[text]	

References

Balshem, H., M. Helfand, H. J. Schunemann, A. D. Oxman, R. Kunz, J. Brozek, G. E. Vist, Y. Falck-Ytter, J. Meerpohl, S. Norris and G. H. Guyatt, 2011: GRADE guidelines: 3. Rating the quality of evidence. *J Clin Epidemiol,* **64,** 401-406.

Borenstein, M., L. V. Hedges, J. P. T. Higgins and H. R. Rothstein, 2009: *Introduction to Meta-Analysis*. John Wiley & Sons, Chichester ; Hoboken.

Gardner, T., 1995: Lyme Disease. In: J. K. Remington, J. (ed.), *Infectious Diseases of the fetus and newborn infant.* W. B. Saunders company.

Gardner, T., 2001: Chapter 11, Lyme Disease. In: J. K. Remington, J. (ed.), *Infectious Diseases of the Fetus and Newborn, 5th ed.* Saunders.

Guyatt, G., A. D. Oxman, E. A. Akl, R. Kunz, G. Vist, J. Brozek, S. Norris, Y. Falck-Ytter, P. Glasziou, H. Debeer, R. Jaeschke, D. Rind, J. Meerpohl, P. Dahm and H. J. Schunemann, 2011a: GRADE guidelines: 1. Introduction-GRADE evidence profiles and summary of findings tables. *J Clin Epidemiol,* **64,** 383-394.

Guyatt, G. H., A. D. Oxman, S. Sultan, P. Glasziou, E. A. Akl, P. Alonso-Coello, D. Atkins, R. Kunz, J. Brozek, V. Montori, R. Jaeschke, D. Rind, P. Dahm, J. Meerpohl, G. Vist, E. Berliner, S. Norris, Y. Falck-Ytter, M. H. Murad and H. J. SchÃ¼nemann, 2011b: GRADE guidelines: 9. Rating up the quality of evidence. *Journal of clinical epidemiology,* **64,** 1311-1316.

Higgins, J. and S. Green, 2011: Cochrane Handbook for Systematic Reviews of Interventions. Cochrane collaboration.

Higgins, J. P. T. and D. G. Altman, 2008: Chapter 8: Assessing risk of bias in included studies. In: G. S. e. In: Higgins Jpt (ed.), *Cochrane Handbook for Systematic Reviews of Interventions Version 5.0.1 (updated September 2008).* The Cochrane Collaboration.

McClure, E. M. and R. L. Goldenberg, 2009: Infection and stillbirth. *Semin Fetal Neonatal Med,* **14,** 182-189.

Mylonas, I., 2011: Borreliosis during pregnancy: a risk for the unborn child? *Vector borne zoonotic dis,* **11,** 891-898.

Schunemann, H., S. Hill, G. Guyatt, E. A. Akl and F. Ahmed, 2011: The GRADE approach and Bradford Hill's criteria for causation. *J Epidemiol Community Health,* **65,** 392-395.

Shapiro, E. D. G., M.A.;, 2011: Chapter 17: Borrelia Infections: Lyme Disease and Relapsing Fever. In: W. Britt (ed.), *Infectious Diseases of the Fetus and Newborn, 7th ed.* Elsevier.

The, G. W. G., 2013: GRADE guidelines - best practices using the GRADE framework.

Walsh, C. A., E. W. Mayer and L. V. Baxi, 2007: Lyme disease in pregnancy: case report and review of the literature. *Obstet Gynecol Surv,* **62,** 41-50.
