# Supplementary material for: A systematic review on the impact of gestational Lyme disease in humans on the fetus and newborn
Source: PLoS One. 2018 Nov 12;13(11):e0207067. doi: 10.1371/journal.pone.0207067 (PMC6231644; doi:10.1371/journal.pone.0207067)
Supplement: S2 Text — (PDF) [file pone.0207067.s002.pdf]

S2: List of 45 references included in the systematic review on the impact of gestational Lyme disease in humans on the fetus and newborn

(1-45)

1. Andrasova V, Svarovsky J, Matousek B. [Lyme disease in pregnancy]. Cesk Gynekol. 1988;53(1):39-41.
2. Bracero LA, Wormser GP, Leikin E, Tejani N. Prevalence of seropositivity to the Lyme disease spirochete during pregnancy in an epidemic area. A preliminary report. . journal of maternal and fetal investigations. 1992;2:265-8.
3. Brzostek T. Human granulocytic ehrlichiosis co-incident with Lyme borreliosis in pregnant woman--a case study. Przegląd epidemiologiczny. 2004;58(2):289-94.
4. Bussen S, Steck T. [Manifestation of Lyme arthritis in the puerperal period]. Z Geburtshilfe Perinatol. 1994;198(4):150-2.
5. Carlomagno G, Luksa V, Candussi G, Magaton Rizzi G, Trevisan G. Lyme Borrelia positive serology associated with spontaneous abortion in an endemic Italian area. Acta Eur Fertil. 1988;19(5):279-81.
6. Ciesielski CAR, H.; Johnson, S.;. Prospective study of pregnancy outcome in women with Lyme disease (abstract). 27th ICAAC1987.
7. Dlesk A, Broste SK, Harkins PG, McCarty PA, Mitchell PD. Lyme seropositivity and pregnancy outcome in the absence of symptoms of Lyme disease. . Arthritis Rheum 1989. p. S46.
8. Figueroa R, Bracero LA, Aguero-Rosenfeld M, Beneck D, Coleman J, Schwartz I. Confirmation of Borrelia burgdorferi spirochetes by polymerase chain reaction in placentas of women with reactive serology for Lyme antibodies. Gynecol Obstet Invest. 1996;41(4):240-3.
9. Gasser R, Dusleag J, Reisinger E, Stauber R, Grisold M, Pongratz S, et al. A most unusual case of a whole family suffering from late Lyme borreliosis for over 20 years. Angiology. 1994;45(1):85-6.
10. Grandsaerd MJ, Meulenbroeks AA. Lyme borreliosis as a cause of facial palsy during pregnancy. Eur J Obstet Gynecol Reprod Biol. 2000;91(1):99-101.
11. Hemels MAC, Gerards LJ, Kwee A, Wolfs TFW. Lyme borreliosis during pregnancy: Consequences for the newborn? Tijdschr Kindergeneeskd. 2002;70(2):65-7.
12. Hercogova J, Tomankova M, Frosslova D, Janovska D. Early stage of Lyme borreliosis during pregnancy: Treatment of 15 women with erythema migrans. CESKO-SLOV GYNEKOL. 1993;58(5):229-32.
13. Hulinska D, Votypka J, Vanousova D, Hercogova J, Hulinsky V, Drevova H, et al. Identification of Anaplasma phagocytophilum and Borrelia burgdorferi sensu lato in patients with erythema migrans. Folia Microbiol (Praha). 2009;54(3):246-56.
14. Isailović G, Veljković M, Soć N, Krstić B, Bjekić M. Erythema migrans after a tick bite in a pregnant woman. Glas Srpska akademija nauka i umetnosti Odeljenje medicinskih nauka. 1993(43):173-5.
15. Jones CR, Smith H, Gibb E, Johnson L. Gestational Lyme Disease Case Studies of 102 Live Births. Lyme Times. 2005(summer 2005):36-8.

16. Jovanovic R, Hajric A, Cirkovic A, Mikovic Z, Dmitrovic R. [Lyme disease and pregnancy]. Glas Srp Akad Nauka [Med]. 1993(43):169-72.

17. Lakos A, Solymosi N. Maternal Lyme borreliosis and pregnancy outcome. Int J Infect Dis. 2010;14(6):e494-8.

18. Lampert F. Infantile multisyslem inflammatory disease: another case of a new syndrome Eur J Pediatr. 1986;144:593-6.

19. Lavoie PE, Lattner BP, Duray PH, Malawista SE, Barbour AG, Johnson RC. Culture positive, seronegative transplacental Lyme borreliosis infant mortality. . Arthritis Rheum; 1987. p. S50.

20. Londero F, San Marco L, Silvestri D, Ruscio M, Bassini D. Lyme disease in pregnancy. Prog Obstet Ginecol. 1998;41(3):146-8.

21. MacDonald AB. Human fetal borreliosis, toxemia of pregnancy, and fetal death. Zentralbl Bakteriol Mikrobiol Hyg [A]. 1986;263(1-2):189-200.

22. MacDonald AB. Gestational Lyme borreliosis. Implications for the fetus. Rheum Dis Clin North Am. 1989;15(4):657-77.

23. MacDonald AB, Benach JL, Burgdorfer W. Stillbirth following maternal Lyme disease. N Y State J Med. 1987;87(11):615-6.

24. Maraspin V, Cimperman J, Lotric-Furlan S, Pleterski-Rigler D, Strle F. Erythema migrans in pregnancy. Wien Klin Wochenschr. 1999;111(22-23):933-40.

25. Maraspin V, Ruzic-Sabljic E, Pleterski-Rigler D, Strle F. Pregnant women with erythema migrans and isolation of borreliae from blood: course and outcome after treatment with ceftriaxone. Diagn Microbiol Infect Dis. 2011;71(4):446-8.

26. Markowitz LE, Steere AC, Benach JL, Slade JD, Broome CV. Lyme disease during pregnancy. Jama. 1986;255(24):3394-6.

27. Mikkelsen AL, Palle C. Lyme disease during pregnancy. Acta Obstet Gynecol Scand. 1987;66(5):477-8.

28. Moniuszko A, Czupryna P, Pancewicz S, Kondrusik M, Penza P, Zajkowska J. Borrelial lymphocytoma-A case report of a pregnant woman. Ticks Tick-borne Dis. 2012;3(4):257-8.

29. Nadal D, Hunziker UA, Bucher HU, Hitzig WH, Duc G. Infants born to mothers with antibodies against Borrelia burgdorferi at delivery. Eur J Pediatr. 1989;148(5):426-7.

30. Nafeev AA. A case of Lyme disease in pregnancy. Zhurnal Mikrobiologii Epidemiologii i Immunobiologii. 2001(3):121-2.

31. O'Brien JM, Baum JD. Low-grade fever, erythematous rash in pregnant woman • Dx? J Fam Pract. 2017;66(8):E9-E10.

32. Önk G, Acun C, Kalayci M, Çağavi F, Açikgöz B, Tanriverdi HA. Gestational lyme disease as a rare cause of congenital hydrocephalus. J Turkish German Gynecol Assoc Artemis. 2005;6(2):156-7.

33. Remy JM, Chevrant-Breton O, Logeais B, Patoux-Pibouin M, Chevrier S, Chevrant-Breton J. Lyme disease during pregnancy: About one case. NOUV DERMATOL. 1994;13(9):682.

34. Schaumann R, Fingerle V, Buchholz K, Spencker FB, Rodloff AC. Facial palsy caused by Borrelia infection in a twin pregnancy in an area of nonendemicity. Clin Infect Dis. 1999;29(4):955-6.

35. Schlesinger PA, Duray PH, Burke BA, Steere AC, Stillman MT. Maternal-fetal transmission of the Lyme disease spirochete, Borrelia burgdorferi. Ann Intern Med. 1985;103(1):67-8.

36. Schutzer SE, Janniger CK, Schwartz RA. Lyme disease during pregnancy. Cutis. 1991;47(4):267-8.

37. Strobino B, Abid S, Gewitz M. Maternal Lyme disease and congenital heart disease: A case-control study in an endemic area. Am J Obstet Gynecol. 1999;180(3 Pt 1):711-6.

38. Strobino BA, Williams CL, Abid S, Chalson R, Spierling P. Lyme disease and pregnancy outcome: a prospective study of two thousand prenatal patients. Am J Obstet Gynecol. 1993;169(2 Pt 1):367-74.

39. Trevisan G, Stinco G, Cinco M. Neonatal skin lesions due to a spirochetal infection: a case of congenital Lyme borreliosis? Int J Dermatol. 1997;36(9):677-80.

40. Troyano-Luque J, Padilla-Perez A, Martinez-Wallin I, Alvarez de la Rosa M, Mastrolia SA, Trujillo JL, et al. Short and long term outcomes associated with fetal cholelithiasis: a report of two cases with antenatal diagnosis and postnatal follow-up. Case Rep Obstet Gynecol. 2014;2014:714271.

41. Walsh CA, Mayer EW, Baxi LV. Lyme disease in pregnancy: case report and review of the literature. Obstet Gynecol Surv. 2007;62(1):41-50.

42. Weber K, Bratzke HJ, Neubert U, Wilske B, Duray PH. Borrelia burgdorferi in a newborn despite oral penicillin for Lyme borreliosis during pregnancy. Pediatr Infect Dis J. 1988;7(4):286-9.

43. Weber K, Neubert U. Clinical features of early erythema migrans disease and related disorders. Zentralbl Bakteriol Mikrobiol Hyg [A]. 1986;263(1-2):209-28.

44. Williams CL, Benach JL, Curran AS, Spierling P, Medici F. Lyme Disease during Pregnancy A Cord Blood Serosurvey. Ann N Y Acad Sci. 1988;539(1):504-6.

45. Williams CL, Strobino B, Weinstein A, Spierling P, Medici F. Maternal Lyme disease and congenital malformations: a cord blood serosurvey in endemic and control areas. Paediatr Perinat Epidemiol. 1995;9(3):320-30.
